# Toxicity profiles of immune checkpoint inhibitors for recurrent or metastatic head and neck squamous cell carcinoma: A systematic review and meta‐analysis

**DOI:** 10.1002/cam4.7119

**Published:** 2024-03-30

**Authors:** Shoutao Dang, Xinyu Li, Heshu Liu, Shuyang Zhang, Wei Li

**Affiliations:** ^1^ Cancer Center, Beijing Tongren Hospital Capital Medical University Beijing China

**Keywords:** head and neck squamous cell carcinoma, immune checkpoint inhibitors, meta‐analysis, treatment‐related adverse events

## Abstract

**Background:**

Immune checkpoint inhibitors (ICIs) are widely used in recurrent or metastatic head and neck squamous cell carcinoma (R/M HNSCC); however, the toxicity profiles are inconclusive.

**Methods:**

Clinical trials evaluating ICIs for R/M HNSCC were searched from online databases. The characteristics of the studies and the results of incidences of any grade treatment‐related adverse events (trAEs), grade three or more trAEs, treatment‐related deaths, trAEs leading to discontinuation of treatment, and specific trAEs were extracted.

**Results:**

Twenty studies with 3756 patients were included. The pooled incidences of any grade trAEs, grade three or more trAEs, treatment‐related deaths, trAEs leading to discontinuation of treatment for overall population were 62.07% (95% CI, 59.07%–65.02%), 13.82% (95% CI, 11.23%–16.62%), 0.39% (95% CI, 0.15%–0.71%), 3.99% (95% CI, 2.36%–5.95%), respectively. Programmed cell death protein 1 (PD‐1) inhibitors monotherapy and ICIs combination therapy had significantly higher incidences of any grade trAEs (odds ratio [OR], 1.25, 95% CI, 1.05–1.49 and 1.36, 95% CI, 1.15–1.60, respectively), grade three or more trAEs (OR, 1.41, 95% CI, 1.08–1.84 and 1.79, 95% CI, 1.39–2.30, respectively), trAEs leading to discontinuation of treatment (OR, 3.98, 95% CI, 2.06–7.70 and 10.14, 95% CI, 5.49–18.70, respectively) compared with programmed death‐ligand 1 (PD‐L1) inhibitors monotherapy. ICIs combination therapy had a significantly higher incidence of grade three or more trAEs compared with PD‐1 inhibitors monotherapy (OR, 1.27, 95% CI, 1.03–1.55); however, the incidences of any grade trAEs and trAEs leading to discontinuation of treatment were not significant different.

**Conclusions:**

Our study suggests that the incidences of grade three or more trAEs, treatment‐related deaths, and trAEs leading to discontinuation of treatment are low in R/M HNSCC patients treated with ICIs. PD‐L1 inhibitors monotherapy may be safer compared with PD‐1 inhibitors monotherapy and ICIs combination therapy.

## 1 INTRODUCTION

Head and neck squamous cell carcinoma (HNSCC) is one of the major causes of cancer‐associated death, with more than 740,000 new cases in the world annually and about 360,000 deaths during the same time period.^1^ Recurrent or metastatic (R/M) HNSCC has a poor prognosis, with a median survival time of less than 12 months.^2^ Immune checkpoint inhibitors (ICIs) have been proven to be effective in a variety of neoplasms. For R/M HNSCC, the overall survival (OS) is also prolonged by programmed cell death protein 1 (PD‐1) inhibitors in platinum‐refractory setting and first‐line setting compared with conventional systemic therapy^3^, ^4^, ^5^; however, negative results are found for the programmed death‐ligand 1 (PD‐L1) inhibitors and dual‐ICIs therapy.^6^, ^7^, ^8^

ICIs are considered to be safe; however, with the increasing use of drugs, the number of immune‐related adverse events (irAEs) also rises. ICIs have a wide spectrum of irAEs, which may involve any organ or system.^9^ Additionally, irAEs may also manifest as late‐onset, persistent, and even life‐threatening.^10^ Furthermore, irAEs assessment can be subjective, with independent clinicians judging inconsistent results of irAEs.^11^ Therefore, the toxicity characteristics of ICIs still require better understanding.

ICIs are currently widely used in a variety of cancer types. Several meta‐analyses have evaluated irAEs for different cancer types and treatment regimens^9^; however, the value of examining HNSCC‐specific irAEs is still unclear. We conducted a meta‐analysis to evaluate the toxicity profiles of ICIs for R/M HNSCC.

## 2 METHODS

### 2.1 Search strategy

This meta‐analysis followed the PRISMA (Preferred Reporting Items for Systematic Reviews and Meta‐Analyses) statement.^12^

We searched the articles from online databases PubMed, Embase, and Cochrane Library up to September 31, 2023. The Medical Subject Headings (MeSH) terms and their entry terms were applied as follows: “nivolumab OR ipilimumab OR immune checkpoint inhibitors OR programmed death‐ligand 1 inhibitor OR PD‐L1 Inhibitor OR cytotoxic T‐lymphocyte‐associated protein 4 inhibitor OR CTLA‐4 inhibitor OR programmed cell death protein 1 inhibitor OR PD‐1 inhibitor OR pembrolizumab OR camrelizumab OR cemiplimab OR durvalumab OR atezolizumab OR tremelimumab OR avelumab” AND “hypopharyngeal neoplasms OR oropharyngeal neoplasms OR laryngeal neoplasms OR mouth Neoplasms OR squamous cell carcinoma of head and neck”.

### 2.2 Inclusion and exclusion criteria

The inclusion criteria were as follows: (a) patients with pathologically confirmed R/M HNSCC; (b) there was at least one group that was treated with ICIs monotherapy or ICIs plus another immunotherapy; (c) there was at least one outcome of the incidences of treatment‐related adverse events (trAEs), treatment‐related deaths, trAEs leading to discontinuation of treatment, and specific trAEs was reported.

The exclusion criteria were as follows: (a) patients with squamous cell carcinoma of the nasal cavity, paranasal sinuses, nasopharynx, or cutaneous; (b) patients with other pathological types; (c) patients treated with ICIs plus radiotherapy, chemotherapy, or targeted therapy; (d) locoregionally advanced HNSCC treated with induction/neoadjuvant ICIs; (e) the study data could not be extracted directly or indirectly; (f) the study was not published in English; (f) retrospective study, conference abstract, case report, comment, review, animal study, and mechanistic study.

### 2.3 Quality assessment

The risk of bias was assessed by two investigators (Shoutao Dang and Xinyu Li) according to the Cochrane Collaboration's tool for randomized controlled trials (RCT) and the Methodological Index for Nonrandomized Studies (MINORS) tool for nonrandomized trials.^13^

### 2.4 Data extraction and statistical analysis

The data were extracted by two investigators (Shoutao Dang and Xinyu Li). The following information were included: first author, publication year, trial phase, study design, sample size, eligible patients, interventions and control group, outcomes of the incidences of trAEs, treatment‐related deaths, trAEs leading to discontinuation of treatment, and specific trAEs. Any disagreement was discussed for consensus.

The pooled incidence results and odds ratio (OR) between groups were calculated by Stata 17. The random‐effects model was used for all results.

## 3 RESULTS

### 3.1 Study and data selection

**FIGURE 1:**
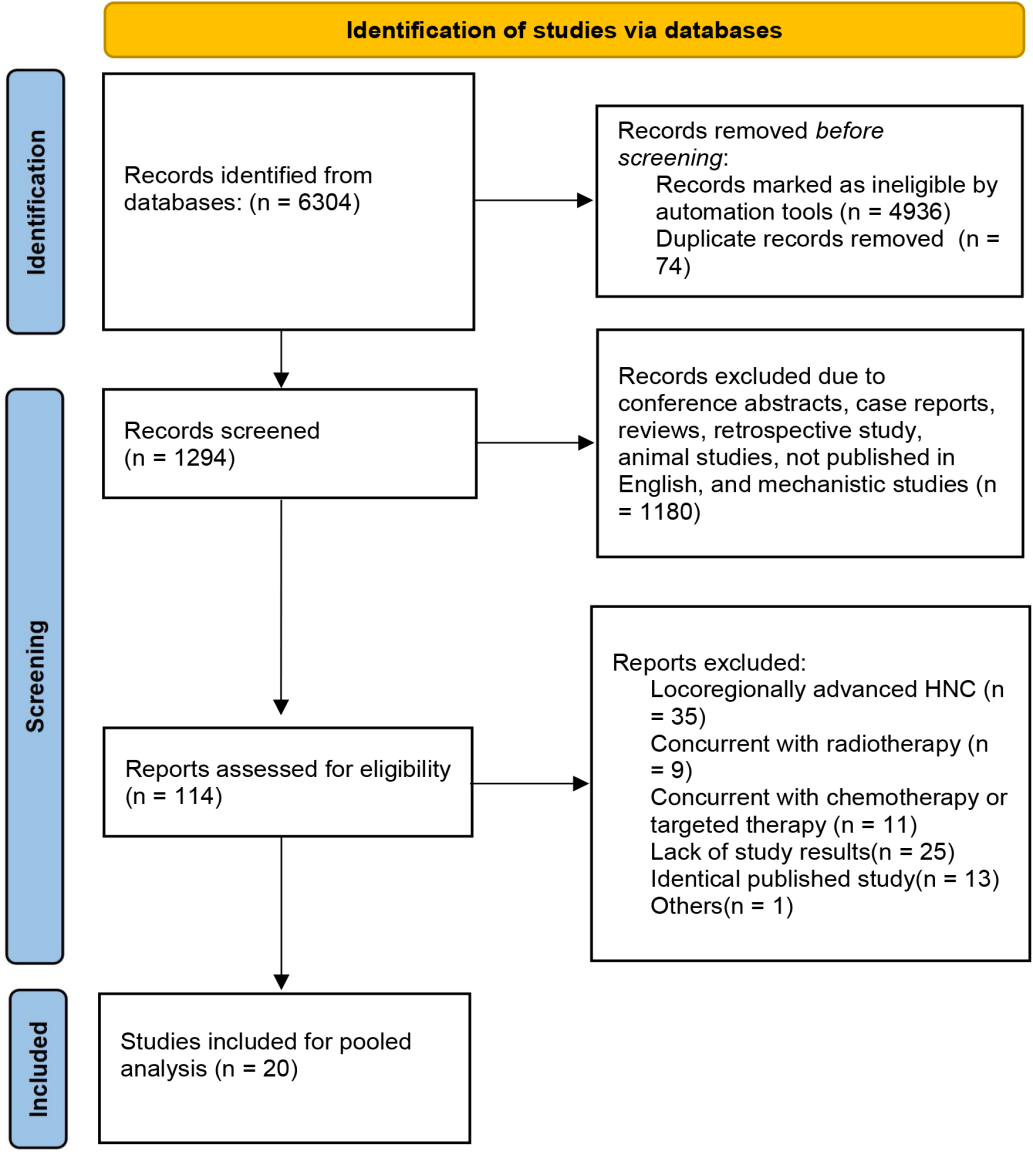
Flow diagram of the screening and selection process.

A total of 6304 records were retrieved from the initial search strategy, among which 4936 were removed when the publication type was limited to clinical trials. Seventy‐four records were excluded for the duplicate. One thousand one hundred and eighty studies were further excluded for abstracts, comments, case reports, reviews, retrospective studies, animal studies, and mechanistic studies. One hundred and fourteen reports were assessed for eligibility, and 94 were excluded (locoregionally advanced disease = 35, concurrent with radiotherapy = 9, concurrent with chemotherapy or targeted therapy = 11, lack of results = 25, identical published study = 13, others = 1). Ultimately, 20 studies with 3756 patients were included in this pooled analysis (Figure 1).

### 3.2 Study characteristics and quality assessment

All 20 studies reported the incidences of any grade and grade three or more trAEs (Table 1). Nine studies were anti‐PD1 monotherapy,^3^, ^4^, ^5^, ^14^, ^15^, ^16^, ^17^, ^18^, ^19^ eight studies were anti‐PDL1 monotherapy,^6^, ^7^, ^20^, ^21^, ^22^, ^23^, ^24^, ^25^ seven studies were ICIs combination therapy,^6^, ^7^, ^8^, ^14^, ^20^, ^26^, ^27^ five studies were median duration of treatment ≤3months, eight studies were >3 months, nine studies were RCT, and 11 studies were non‐RCT. Seventeen studies reported the incidence of treatment‐related deaths, among which eight studies were anti‐PD1 monotherapy, eight studies were anti‐PDL1 monotherapy, and six studies were ICIs combination therapy. Sixteen studies reported the incidence of trAEs leading to discontinuation of treatment, among which six studies were anti‐PD1 monotherapy, seven studies were anti‐PDL1 monotherapy, and seven studies were ICIs combination therapy. Thirty‐two specific trAEs were reported in at least two studies.

**TABLE 1:** The characteristic of the studies included for the meta‐analysis.

Author	Year	Study type	Eligible patients	Intervention	Median duration of treatment	Number of patients
R.L. Ferris	2016	Phase 3 RCT	Platinum‐refractory R/M HNSCC	Nivolumab	1.9 months	236
Ezra E. W. Cohen	2019	Phase 3 RCT	Platinum‐refractory R/M HNSCC	Pembrolizumab	NA	246
Barbara Burtness	2019	Phase 3 RCT	First‐line R/M HNSCC	Pembrolizumab	3.5 months	300
R. L. Ferris	2020	Phase 3 RCT	Platinum‐refractory R/M HNSCC	Durvalumab Durvalumab + tremelimumab	3.2 months 2.8 months	237 246
Robert I. Haddad	2023	Phase 3 RCT	First‐line R/M HNSCC	Nivolumab + ipilimumab	3.8 months	468
A. Psyrri	2023	Phase 3 RCT	First‐line R/M HNSCC	Durvalumab Durvalumab + tremelimumab	3.7 months 3.7 months	202 408
Lillian L. Siu	2019	Phase 2 RCT	PD‐L1 low/negative platinum‐refractory R/M HNSCC	Durvalumab + tremelimumab Durvalumab	NA	133 65
Matthew H. Taylor	2022	Phase 2 RCT	Platinum‐refractory R/M HNSCC	Pembrolizumab	3.5 months	39
Kevin J. Harrington	2023	Phase 2 RCT	Platinum‐refractory and platinum‐eligible R/M HNSCC	Nivolumab + ipilimumab Nivolumab	2.7 months (Platinum‐refractory) 1.9 months (Platinum‐eligible) 3.4 months (Platinum‐refractory) 3.7 months (Platinum‐eligible)	280 143
Joshua Bauml	2017	Phase 2	Platinum and cetuximab refractory R/M HNSCC	Pembrolizumab	90 days	171
Dan P. Zandberg	2019	Phase 2	Tumor cell PD‐L1 Expression ≥ 25% platinum‐refractory R/M HNSCC	Durvalumab	3.5 months	111
Neil H. Segal	2019	Phase 1/2	R/M HNSCC	Durvalumab	Six doses Q2w	62
M. Simonelli	2022	Phase 1/2	Platinum‐refractory recurrent HNSCC	Isatuximab + atezolizumab	NA	29
Glenn J. Hanna	2022	Phase 2	Recurrent resectable HNSCC	Nivolumab + lirilumab	NA	28
Jennifer L. Leddon	2022	Phase 2	Recurrence HNSCC	Nivolumab	NA	39
Tanguy Y. Seiwert	2016	Phase 1b	R/M HNSCC	Pembrolizumab	NA	60
A. D. Colevas	2018	Phase 1	Unresectable or incurable HNC	Atezolizumab	3.4 months	32
Kevin J. Harrington	2020	Phase 1b	Platinum‐refractory R/M HNSCC	Intratumoral T‐VEC + pembrolizumab	NA	36
Byoung Chul Cho	2020	Phase 1	Platinum‐refractory R/M HNSCC	Bintrafusp alfa	NA	32
Joël Guigay	2021	Phase 1b	Platinum ineligible/ refractory R/M HNSCC	Avelumab	NA	153

Abbreviation: NA, not applicable.

The nine RCT studies were of high quality and mostly at low risk of bias, and the other 11 non‐RCT studies without the control group scored 12–15 of 16 on MINORS (Figure S1; Table S1).

### 3.3 Pooled incidence of any grade trAEs

**FIGURE 2:**
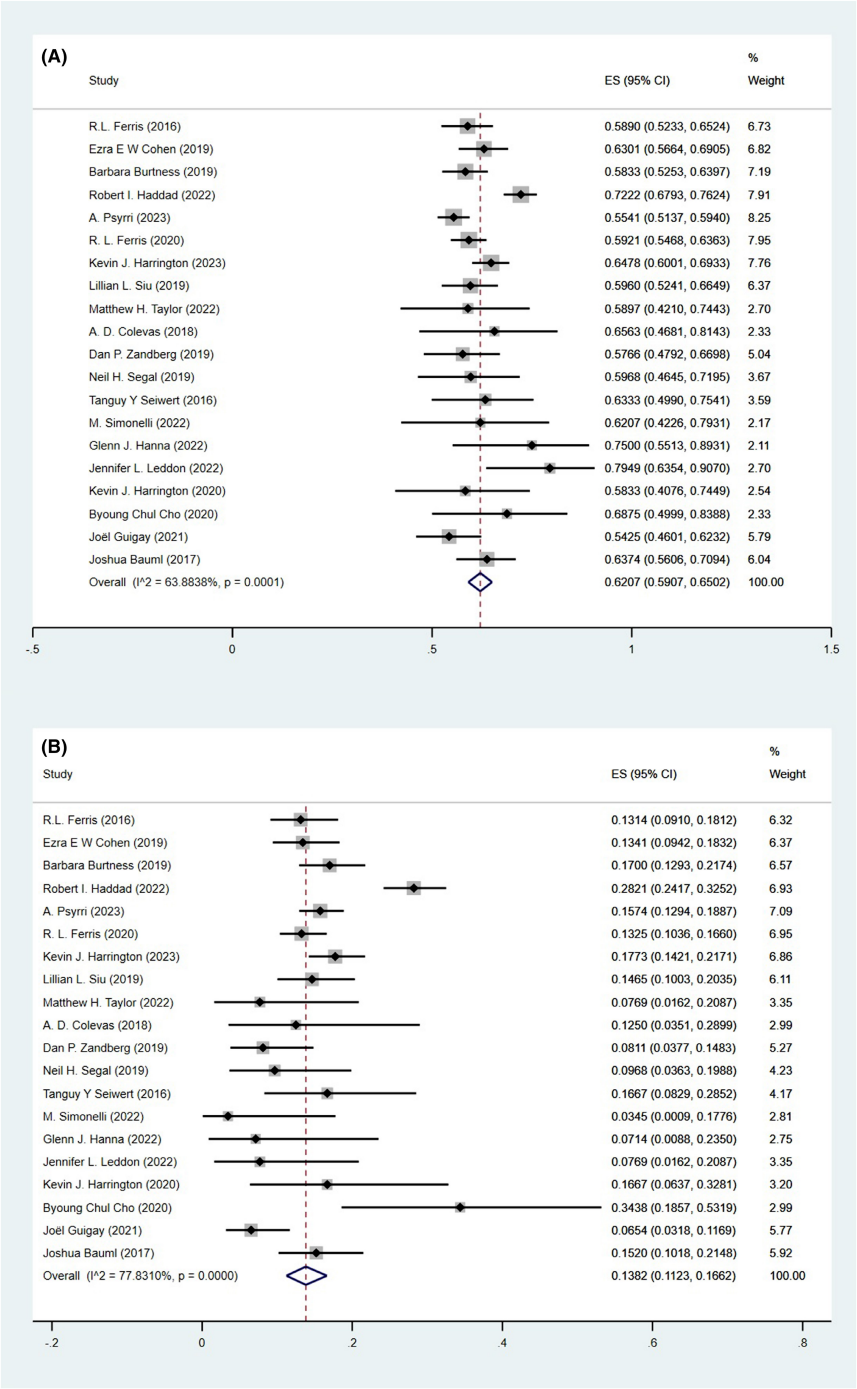
Pooled incidences of overall population: any grade trAEs (A), grade three or more trAEs (B).

The pooled incidences of any grade trAEs were 62.07% (95% CI, 59.07%–65.02%, Figure 2A) for overall population, 62.30% (95% CI, 59.12%–65.43%, Figure S2A) for patients treated with PD‐1 inhibitors monotherapy, 56.92% (95% CI, 51.89%–61.89%, Figure S2B) for patients treated with PD‐L1 inhibitors monotherapy, 64.20% (95% CI, 59.10%–69.14%, Figure S2C) for patients treated with ICIs combination therapy, 61.74% (95% CI, 58.68%–64.75%, Figure S2D) for whose median duration of treatment ≤3 months, 61.53% (95% CI, 55.72%–67.19%, Figure S2E) for whose median duration of treatment >3 months, 61.46% (95% CI, 57.36%–65.48%, Figure S2F) for RCT studies, and 62.59% (95% CI, 58.25%–66.84%, Figure S2G) for non‐RCT studies.

PD‐1 inhibitors monotherapy (OR, 1.25, 95% CI, 1.05–1.49) and ICIs combination therapy (OR, 1.36, 95% CI, 1.15–1.60) had significantly higher incidences of any grade trAEs compared with PD‐L1 inhibitors monotherapy; however, there was no significant difference for ICIs combination versus PD‐1 inhibitors monotherapy (OR, 1.09, 95% CI, 0.93–1.27), median duration of treatment ≤3 months versus >3 months (OR, 1.01, 95% CI, 0.86–1.18), and RCT studies versus non‐RCT studies (OR, 0.96, 95% CI, 0.81–1.13, Table 2).

**TABLE 2:** Subgroup analysis of any grade trAEs, grade three or more trAEs, treatment‐related deaths, and trAEs leading to discontinuation.

	Population	Pooled incidence (95% CI)	Population	Pooled incidence (95% CI)	OR (95% CI)	*p*‐value
Any grade trAEs	PD‐1	62.30% (59.12%–65.43%)	PD‐L1	56.92% (51.89%–61.89%)	1.25 (1.05–1.49)	0.01
	Combination	64.20% (59.10%–69.14%)	PD‐L1	56.92% (51.89%–61.89%)	1.36 (1.15–1.60)	0.0004
	Combination	64.20% (59.10%–69.14%)	PD‐1	62.30% (59.12%–65.43%)	1.09 (0.93–1.27)	0.29
	Duration ≤3 months	61.74% (58.68%–64.75%)	Duration >3 months	61.53% (55.72%–67.19%)	1.01 (0.86–1.18)	0.93
	RCT studies	61.46% (57.36%–65.48%)	Non‐RCT studies	62.59% (58.25%–66.84%)	0.96 (0.81–1.13)	0.59
Grade three or more trAEs	PD‐1	14.12% (12.21%–16.13%)	PD‐L1	10.41% (7.27%–14.00%)	1.41 (1.08–1.84)	0.01
	Combination	17.20% (12.66%–22.26%)	PD‐L1	10.41% (7.27%–14.00%)	1.79 (1.39–2.30)	<0.00001
	Combination	17.20% (12.66%–22.26%)	PD‐1	14.12% (12.21%–16.13%)	1.27 (1.03–1.55)	0.02
	Duration ≤3 months	15.50% (12.76%–18.45%)	Duration >3 months	14.33% (9.76%–19.58%)	1.09 (0.88–1.36)	0.41
	RCT studies	15.98% (12.72%–19.52%)	Non‐RCT studies	11.37% (7.74%–15.52%)	1.48 (1.16–1.88)	0.0018
Treatment‐related deaths	PD‐1	0.48% (0.09%–1.07%)	PD‐L1	0.16% (0.00%–0.72%)	2.18 (0.44–10.85)	0.34
	Combination	0.62% (0.09%–1.46%)	PD‐L1	0.16% (0.00%–0.72%)	2.87 (0.63–13.14)	0.17
	Combination	0.62% (0.09%–1.46%)	PD‐1	0.48% (0.09%–1.07%)	1.31 (0.48–3.63)	0.6
trAEs leading to discontinuation	PD‐1	5.32% (2.86%–8.39%)	PD‐L1	1.37% (0.33%–2.89%)	3.98 (2.06–7.70)	<0.0001
	Combination	5.45% (2.98%–8.54%)	PD‐L1	1.37% (0.33%–2.89%)	10.14 (5.49–18.70)	<0.00001
	Combination	5.45% (2.98%–8.54%)	PD‐1	5.32% (2.86%–8.39%)	1.03 (0.69–1.53)	0.89

### 3.4 Pooled incidence of grade three or more trAEs

The pooled incidences of grade three or more trAEs were 13.82% (95% CI, 11.23%–16.62%, Figure 2B) for overall population, 14.12% (95% CI, 12.21%–16.13%, Figure S3A) for patients treated with PD‐1 inhibitors monotherapy, 10.41% (95% CI, 7.27%–14.00%, Figure S3B) for patients treated with PD‐L1 inhibitors monotherapy, 17.20% (95% CI, 12.66%–22.26%, Figure S3C) for patients treated with ICIs combination therapy, 15.50% (95% CI, 12.76%–18.45%, Figure S3D) for whose median duration of treatment ≤3 months, 14.33% (95% CI, 9.76%–19.58%, Figure S3E) for whose median duration of treatment >3 months, 15.98% (95% CI, 12.72%–19.52%, Figure S3F) for RCT studies, and 11.37% (95% CI, 7.74%–15.52%, Figure S3G) for non‐RCT studies.

ICIs combination therapy had a significantly higher incidence of grade three or more trAEs compared with PD‐L1 inhibitors monotherapy (OR, 1.79, 95% CI, 1.39–2.30) and PD‐1 inhibitors monotherapy (OR, 1.27, 95% CI, 1.03–1.55). PD‐1 inhibitors monotherapy also had a significantly higher incidence of grade three or more trAEs compared with PD‐L1 inhibitors monotherapy (OR, 1.41, 95% CI, 1.08–1.84). The pooled incidences of grade three or more trAEs were significantly higher in the RCT studies compared with non‐RCT studies (OR, 1.48, 95% CI, 1.16–1.88). There was no significant difference for median duration of treatment ≤3 months versus >3 months (OR, 1.09, 95% CI, 0.88–1.36, Table 2).

### 3.5 Pooled incidence of treatment‐related deaths

**FIGURE 3:**
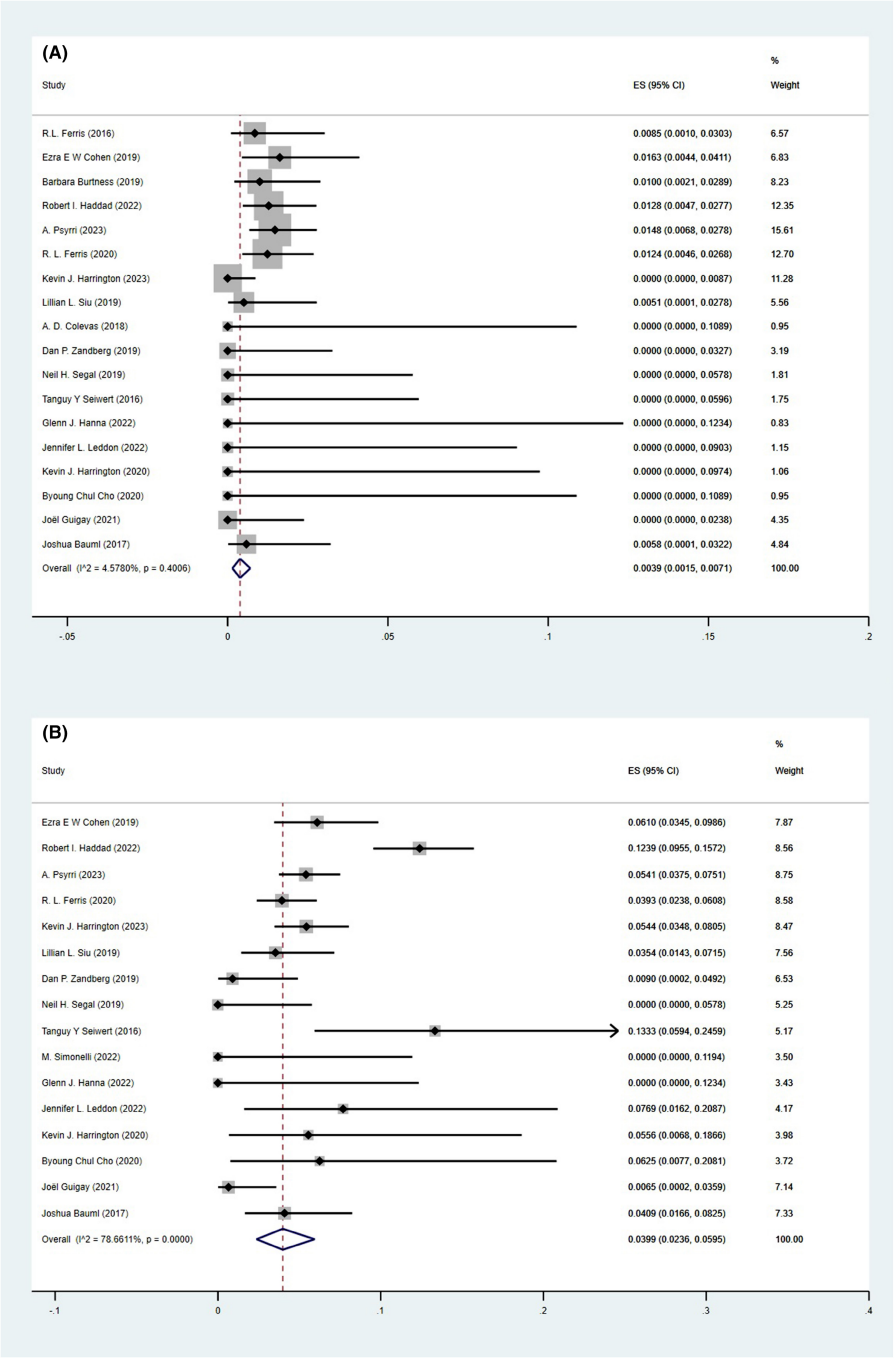
Pooled incidences of overall population: treatment‐related deaths (A), trAEs leading to discontinuation of treatment (B).

The pooled incidences of treatment‐related deaths were 0.39% (95% CI, 0.15%–0.71%, Figure 3A) for overall population, 0.48% (95% CI, 0.09%–1.07%, Figure S4A) for patients treated with PD‐1 inhibitors monotherapy, 0.16% (95% CI, 0.00%–0.72%, Figure S4B) for patients treated with PD‐L1 inhibitors monotherapy, 0.62% (95% CI, 0.09%–1.46%, Figure S4C) for patients treated with ICIs combination therapy.

PD‐L1 inhibitors monotherapy seemed to have a relatively lower incidence of treatment‐related deaths, while ICIs combination therapy seemed to have a relatively higher incidence of treatment‐related deaths; however, no significant differences were found (PD‐1 versus PD‐L1, OR, 2.18, 95% CI, 0.44–10.85; combination versus PD‐L1, OR, 2.87, 95% CI, 0.63–13.14; combination versus PD‐1, OR, 1.31, 95% CI, 0.48–3.63, Table 2).

### 3.6 Pooled incidence of trAEs leading to discontinuation of treatment

The pooled incidences of trAEs leading to discontinuation of treatment were 3.99% (95% CI, 2.36%–5.95%, Figure 3B) for overall population, 5.32% (95% CI, 2.86%–8.39%, Figure S5A) for patients treated with PD‐1 inhibitors monotherapy, 1.37% (95% CI, 0.33%–2.89%, Figure S5B) for patients treated with PD‐L1 inhibitors monotherapy, 5.45% (95% CI, 2.98%–8.54%, Figure S5C) for patients treated with ICIs combination therapy.

PD‐1 inhibitors monotherapy (OR, 3.98, 95% CI, 2.06–7.70) and ICIs combination therapy (OR, 10.14, 95% CI, 5.49–18.70) had significantly higher incidences of trAEs leading to discontinuation of treatment compared with PD‐L1 inhibitors monotherapy; however, there was no significant difference for ICIs combination versus PD‐1 inhibitors monotherapy (OR, 1.03, 95% CI, 0.69–1.53, Table 2).

### 3.7 Pooled incidence of specific trAEs

**FIGURE 4:**
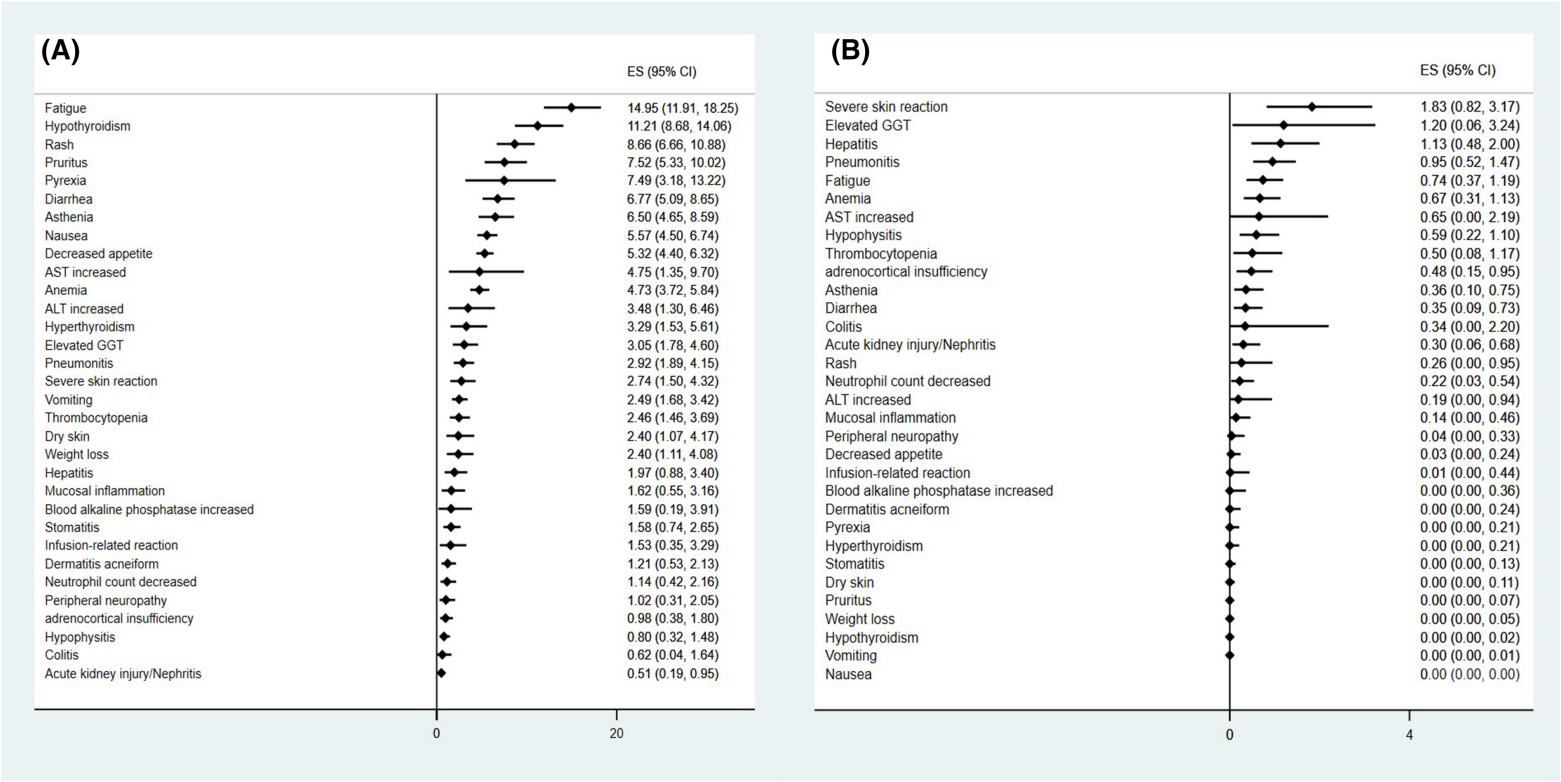
Pooled incidences of specific trAEs reported in at least two studies: any grade (A), grade three or more (B).

Pooled incidences of specific trAEs reported in at least two studies are shown in Figure 4. Fatigue and hypothyroidism were the very common (≥10%) any grade trAEs with pooled incidences as 14.95% (95% CI, 11.91%–18.25%) and 11.24% (95% CI, 8.68%–14.06%), respectively. Rash, pruritus, pyrexia, diarrhea, asthenia, nausea, decreased appetite, aspartate transaminase (AST) increased, anemia, alanine aminotransferase (ALT) increased, hyperthyroidism, elevated gamma‐glutamyl transferase (GGT), pneumonitis, severe skin reaction, vomiting, thrombocytopenia, dry skin, weight loss, hepatitis, mucosal inflammation, blood alkaline phosphatase increased, stomatitis, infusion‐related reaction, dermatitis acneiform, neutrophil count decreased, and peripheral neuropathy were the common (<10%, ≥1%) any grade trAEs; however, adrenocortical insufficiency, hypophysitis, colitis, and acute kidney injury/nephritis were the uncommon (<1%, ≥0.1%) any grade trAEs (Figure 4A; Figure S6).

Severe skin reaction, elevated GGT, and hepatitis were the common trAEs for grade three or more (Figure 4B; Figure S7). Pneumonitis, fatigue, anemia, AST increased, hypophysitis, thrombocytopenia, adrenocortical insufficiency, asthenia, diarrhea, colitis, acute kidney injury/nephritis, rash, neutrophil count decreased, ALT increased, and mucosal inflammation were the uncommon trAEs for grade three or more; however, the other reported specific trAEs were rare (<0.1%, ≥0.01%) or very rare (<0.01%).

## 4 DISCUSSION

ICIs were widely used for R/M HNSCC patients; however, the safety results varied by study. Our meta‐analysis indicated that the pooled incidences of any grade trAEs, grade three or more trAEs, treatment‐related deaths, and trAEs leading to discontinuation of treatment for overall population were 62.07%, 13.82%, 0.39%, 3.99%, respectively; PD‐L1 inhibitors monotherapy had significantly lower incidences of any grade trAEs, grade three or more trAEs, and trAEs leading to discontinuation of treatment compared with both PD‐1 inhibitors monotherapy and ICIs combination therapy; ICIs combination therapy had a significantly higher incidence of grade three or more trAEs compared with PD‐1 inhibitors monotherapy; RCT studies reported a significantly higher incidence of grade three or more trAEs compared with non‐RCT studies. To our knowledge, this is the first meta‐analysis that reports these toxicity results for R/M HNSCC.

Our pooled incidences of trAEs for R/M HNSCC were not fully consistent with some other cancer types. For instance, a meta‐analysis which included advanced esophageal cancer patients reported that the pooled incidences of any grade trAEs were 88% for PD‐1 inhibitors alone population, 79.5% for PD‐1 plus cytotoxic T‐lymphocyte‐associated protein 4 (CTLA‐4) inhibitors population, and the incidences of grade three or more trAEs were 24% for PD‐1 inhibitors alone population, 34.2% for PD‐1 plus CTLA‐4 inhibitors population.^28^ However, another meta‐analysis which included urologic cancer patients showed that the pooled incidences of any grade trAEs were 69.8% for single ICIs therapy, 88.5% for dual‐ICIs therapy, and the incidences of grade three or more trAEs were 16.9% for single ICIs therapy, 41.8% for dual‐ICIs therapy.^29^ Additionally, our pooled incidences of trAEs were much higher compared with previously reported irAEs. A meta‐analysis which included multiple tumor sites reported that the pooled incidences of irAEs were 26.82% for any grade and 6.10% for severe grade in patients treated with PD1/PDL1 inhibitors monotherapy.^30^ ICIs should have a higher incidence of trAEs than irAEs indeed. However, it is hard to say whether there are so many ICIs drug‐related but not immune‐related adverse events or the incidence of irAEs is underestimated. A uniform criterion to evaluate ICIs‐related adverse events is still needed.

Although many patients had trAEs during the ICIs treatment, our pooled incidence of grade three or more trAEs was low. Consequently, the pooled incidences of treatment‐related deaths and trAEs leading to discontinuation of treatment for R/M HNSCC were acceptable. Therefore, our meta‐analysis suggests that ICIs therapy is a safe and tolerable treatment for R/M HNSCC.

Thirty‐two specific trAEs were reported in at least two studies. Fatigue, hypothyroidism, rash, pruritus, and pyrexia were the top five common any grade trAEs. And severe skin reaction, elevated GGT, hepatitis, pneumonitis, and fatigue were the top five common grade three or more trAEs. However, the pooled incidences of these specific trAEs were heterogeneous. Firstly, most trials only reported relatively more frequency trAEs or important trAEs instead of all, so some unreported low‐incidence trAEs were not included in the meta‐analysis. Additionally, there might also be heterogeneity in the diagnosis of trAEs. For instance, increased ALT/AST was reported in some studies, while hepatitis was reported in some other studies. Furthermore, the terms of specific irAEs might be different, too. In one study, 510 irAEs terms were identified from drug labels, but only 156 terms (30.59%) were covered by the Common Terminology Criteria for Adverse Events (CTCAE).^31^ The unique terms of irAEs for the new CTCAE are urgently required, and the Society for Immunotherapy of Cancer (SITC) is making efforts for the upcoming CTCAE v6.0.^10^

Whether the incidence of adverse events varies by ICIs type is still inconclusive. A meta‐analysis which included 20,128 patients from 125 clinical trials suggested that PD‐L1 inhibitors appeared to have a lower incidence of grade three or higher adverse events compared with PD‐1 inhibitors, while the incidence of any grade adverse events was not significantly different.^32^ Another meta‐analysis indicated that the incidences of any grade and grade three or higher trAEs were not significantly different between PD‐1 inhibitors monotherapy and PD‐L1 inhibitors monotherapy in urologic cancer patients; however, dual‐ICIs therapy had significantly higher incidences of any grade and grade three or higher trAEs compared with single ICIs therapy.^29^ Our pooled results showed that ICIs combination therapy had a higher incidence of grade three or more trAEs compared with PD‐1 inhibitors monotherapy in R/M HNSCC patients. Similar results were found in another meta‐analysis with only RCT studies included.^33^ Our pooled results also suggested that PD‐L1 inhibitors monotherapy had significantly lower incidences of any grade trAEs, grade three or more trAEs, and trAEs leading to discontinuation of treatment compared with both PD‐1 inhibitors monotherapy and ICIs combination therapy. In addition to PD‐L1, PD‐L2 was described as a second ligand for PD‐1 with overlapping functions.^34^ PD‐L2 expression in tumor cells was found to be more common in HNSCC samples compared with some other cancer types such as renal cell carcinoma and melanoma.^35^ Therefore, the incomplete blocking of checkpoint signaling due to the high prevalence of PD‐L2 expression in HNSCC might be one of the reasons for the lower toxicity of PD‐L1 inhibitors. PD‐L1 inhibitors might be an option for specific populations such as old or autoimmune disease settings, although they did not significantly improve OS in R/M HNSCC patients.

We did an exploratory analysis for the relationship between the duration of ICIs treatment and the incidence of trAEs, and no significant difference was found between the median duration of treatment ≤3 months and >3 months. PD‐1/PD‐L1 inhibitors seemed to be less dose‐dependent than CTLA‐4 inhibitors.^36^ Additionally, most irAEs occurred within 3 months of treatment initiation.^37^ Therefore, PD‐1/PD‐L1 inhibitors appear to have less accumulative toxicity than chemotherapy. They should be safe for long‐term use, although the optimal treatment duration time of ICIs is still unclear. We also found that the pooled incidence of grade three or more trAEs was significantly higher in the RCT studies. Compared with non‐RCT studies, the RCT studies in this meta‐analysis included many more patients (3003 vs. 753) and registered relatively later, which might be the reasons for this difference. And this difference also suggested that the trAEs of ICIs might be underestimated in some trials.

The pathophysiological mechanisms of irAEs are still unclear,^10^ and irAEs lack standard diagnostic criteria; hence, the irAEs results may vary by investigator. Additionally, some trials applied other terminologies of irAEs, such as adverse events of special interest and immune‐mediated adverse events.^38^ Furthermore, it was difficult to distinguish an adverse event from immune‐related to treatment‐related if ICIs were used in combination with other anti‐cancer therapies such as chemotherapy or targeted therapy. These heterogeneous irAEs data from different trials and investigators might have some influence on the pooled analysis results. Treatment‐related adverse event is a reliable indicator for drug‐related adverse reactions, and it is widely used in clinical trials. Our study included the patients treated with ICIs monotherapy or ICIs plus another immunotherapy drug so that the trAEs results might be a good indicator for toxicity profiles of ICIs.

Our study had limitations. Firstly, there were considerable heterogeneities in the majority of our pooled results, and the sources of heterogeneity were still unclear. Additionally, the comparison between different subgroups was not head‐to‐head. Further studies are needed to confirm these results.

## 5 CONCLUSIONS

The pooled incidences of any grade trAEs, grade three or more trAEs, treatment‐related deaths, and trAEs leading to discontinuation of treatment for the overall population were 62.07%, 13.82%, 0.39%, 3.99%, respectively; PD‐L1 inhibitors monotherapy had significantly lower incidences of any grade trAEs, grade three or more trAEs, and trAEs leading to discontinuation of treatment compared with both PD‐1 inhibitors monotherapy and ICIs combination therapy; ICIs combination therapy had a significantly higher incidence of grade three or more trAEs compared with PD‐1 inhibitors monotherapy.

## AUTHOR CONTRIBUTIONS

**Shoutao Dang:** Conceptualization (equal); data curation (lead); formal analysis (lead); methodology (equal); software (lead); writing – original draft (lead); writing – review and editing (supporting). **Xinyu Li:** Conceptualization (equal); data curation (supporting); formal analysis (supporting); methodology (equal); software (supporting). **Heshu Liu:** Conceptualization (equal); data curation (supporting); formal analysis (supporting); methodology (equal). **Shuyang Zhang:** Conceptualization (equal); methodology (equal). **Wei Li:** Conceptualization (equal); data curation (supporting); formal analysis (supporting); methodology (equal); software (supporting); writing – original draft (supporting); writing – review and editing (lead).

## FUNDING INFORMATION

No specific funding was disclosed.

## CONFLICT OF INTEREST STATEMENT

No potential conflict of interest was reported by the authors.

## Supporting information


Data S1.

Data S2.

Data S3.

## Data Availability

The data underlying this article are available in the article and in its supplementary material.
