# Supplementary material for: Toxicity profiles of immune checkpoint inhibitors for recurrent or metastatic head and neck squamous cell carcinoma: A systematic review and meta‐analysis
Source: Cancer Med. 2024 Mar 30;13(7):e7119. doi: 10.1002/cam4.7119 (PMC10980932; doi:10.1002/cam4.7119)
Supplement: Supplementary file 1 — Data S1. [file CAM4-13-e7119-s003.docx]

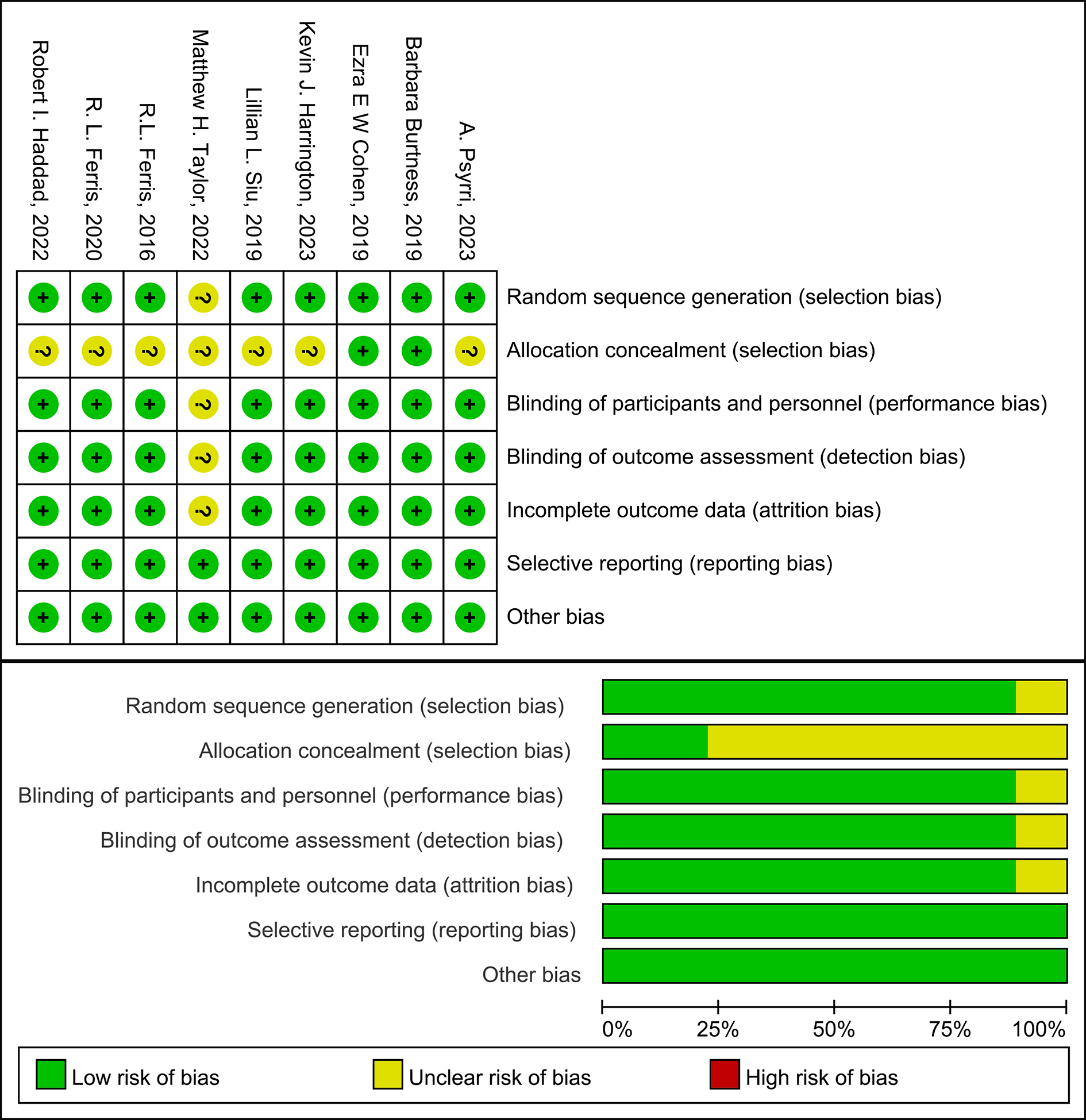


Figure S1. Risk of bias summary and risk of bias graph for RCT studies.

|  | A clearly stated aim | Inclusion of consecutive patients | Prospective collection of data | Endpoints appropriate to the aim of the study | Unbiased assessment of the study endpoint | Follow-up period appropriate | Loss to follow up less than 5% | Prospective calculation of the study size | Total scores |
| --- | --- | --- | --- | --- | --- | --- | --- | --- | --- |
| Joshua Bauml  2017 | 2 | 2 | 2 | 2 | 2 | 2 | 1 | 1 | 14 |
| Dan P. Zandberg  2019 | 2 | 2 | 2 | 2 | 2 | 2 | 1 | 1 | 14 |
| Neil H. Segal  2019 | 2 | 2 | 2 | 2 | 2 | 2 | 1 | 1 | 14 |
| M. Simonelli  2022 | 2 | 2 | 2 | 1 | 2 | 1 | 1 | 1 | 12 |
| Glenn J. Hanna  2022 | 2 | 2 | 2 | 2 | 2 | 2 | 1 | 1 | 14 |
| Jennifer L. Leddon  2022 | 2 | 2 | 2 | 2 | 2 | 2 | 1 | 2 | 15 |
| Tanguy Y Seiwert  2016 | 2 | 2 | 2 | 2 | 2 | 2 | 1 | 2 | 15 |
| A. D. Colevas  2018 | 2 | 2 | 2 | 2 | 2 | 2 | 1 | 1 | 14 |
| Kevin J. Harrington  2020 | 2 | 2 | 2 | 2 | 2 | 2 | 1 | 1 | 14 |
| Byoung Chul Cho  2020 | 2 | 2 | 2 | 2 | 2 | 2 | 1 | 1 | 14 |
| Joël Guigay  2021 | 2 | 2 | 2 | 2 | 2 | 2 | 1 | 1 | 14 |

Table S1. MINORS assessment for Non-RCT studies.


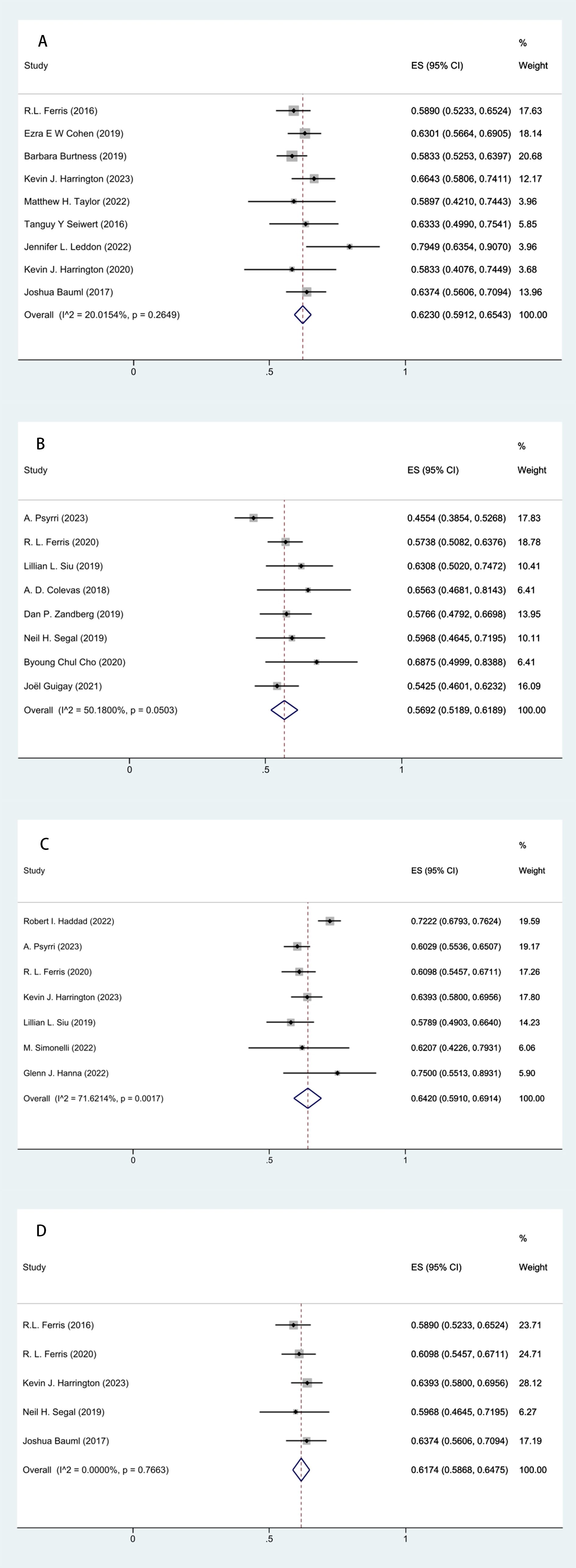


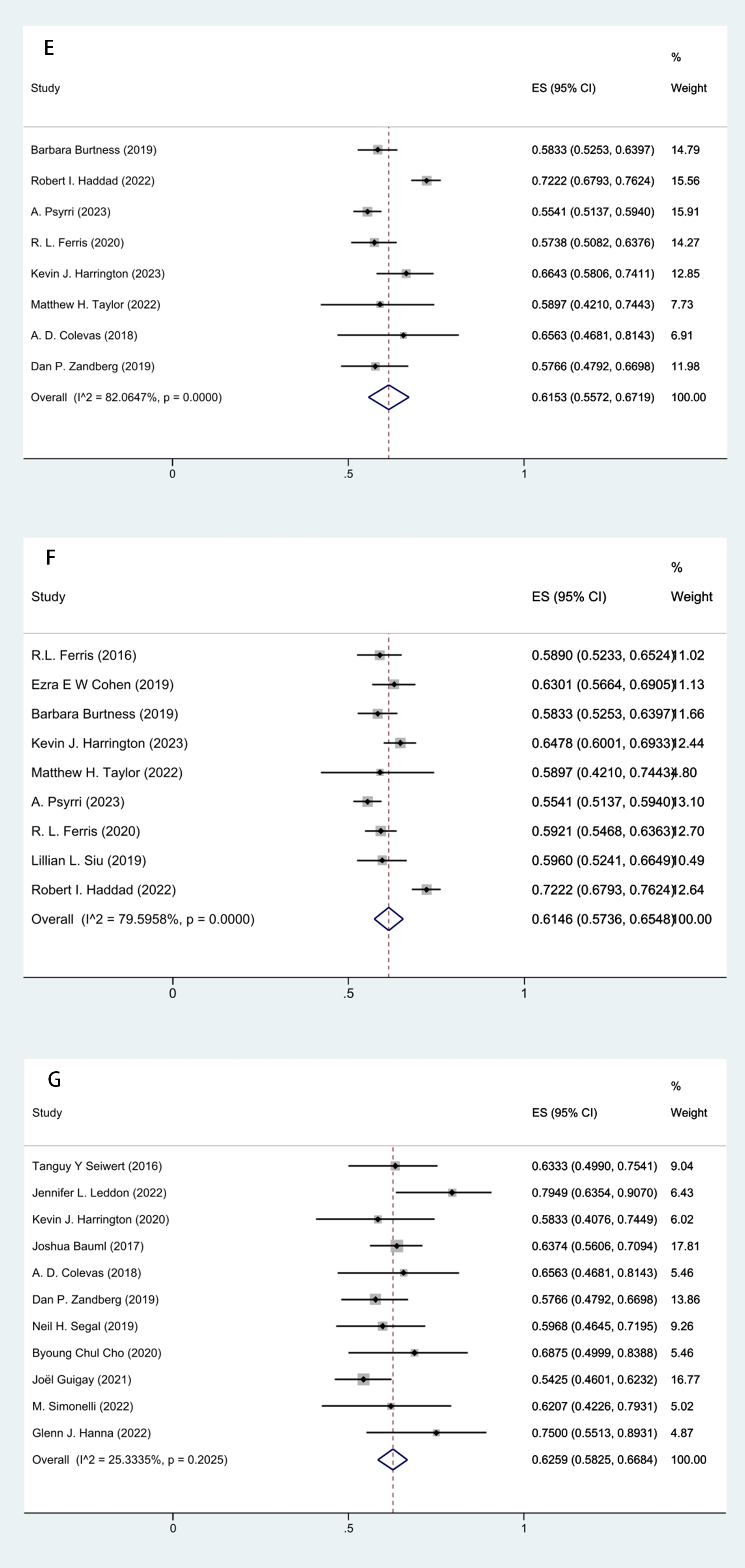


Figure S2. Pooled incidences of any grade trAEs: patients treated with PD-1 (A), patients treated with PD-L1 (B), patients treated with combination therapy (C), median duration of treatment ≤3 months (D), median duration of treatment >3 months (E), RCT studies (F), non-RCT studies (G).


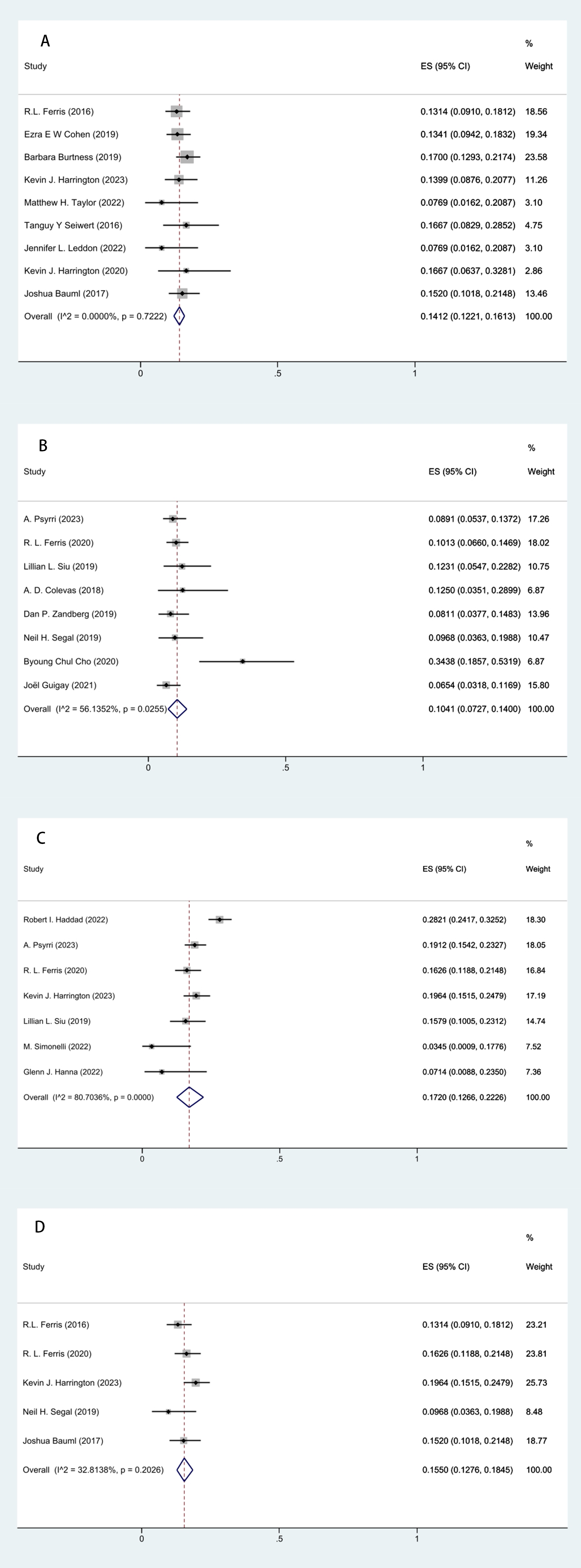


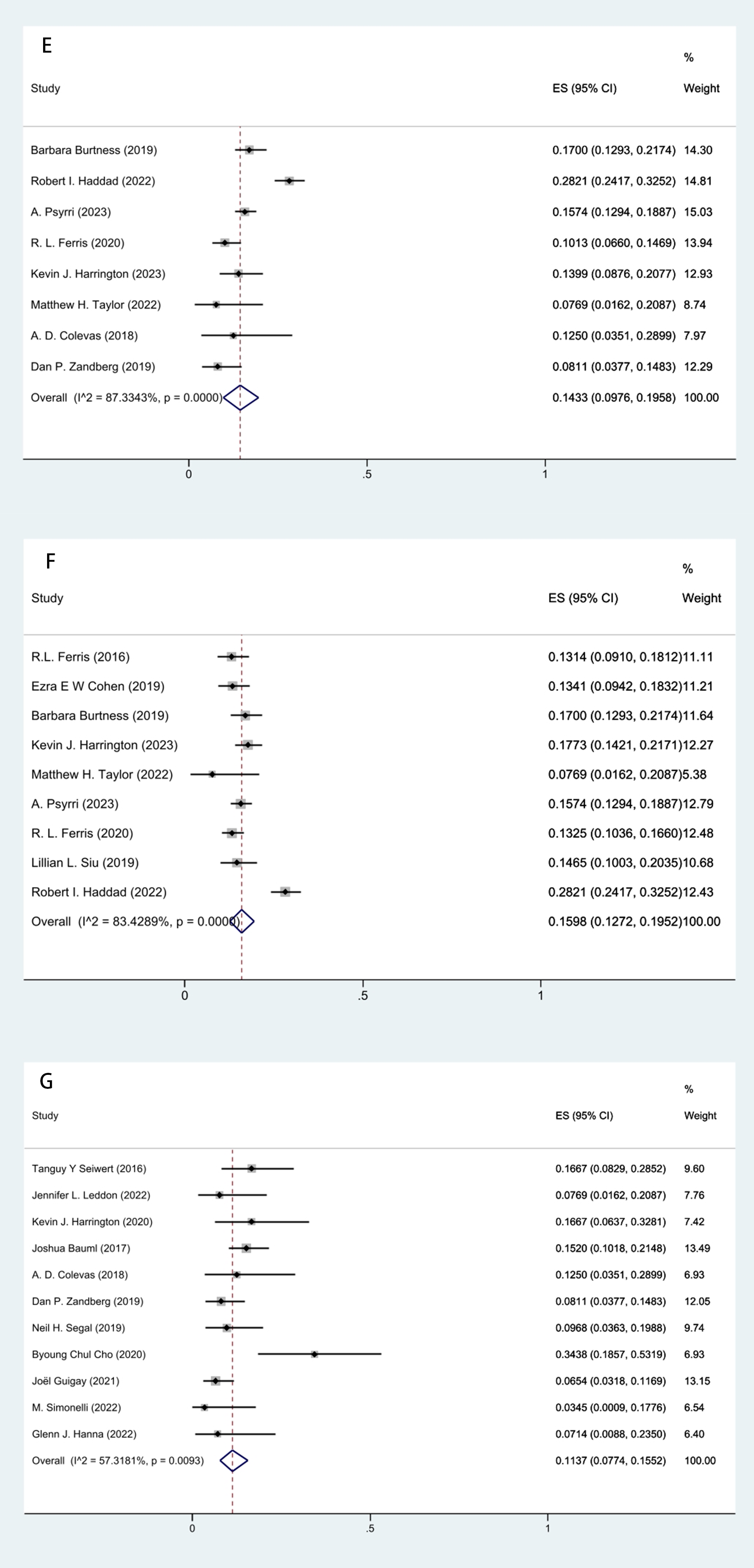


Figure S3. Pooled incidences of grade 3 or more trAEs: patients treated with PD-1 (A), patients treated with PD-L1 (B), patients treated with combination therapy (C), median duration of treatment ≤3 months (D), median duration of treatment >3 months (E), RCT studies (F), non-RCT studies (G).


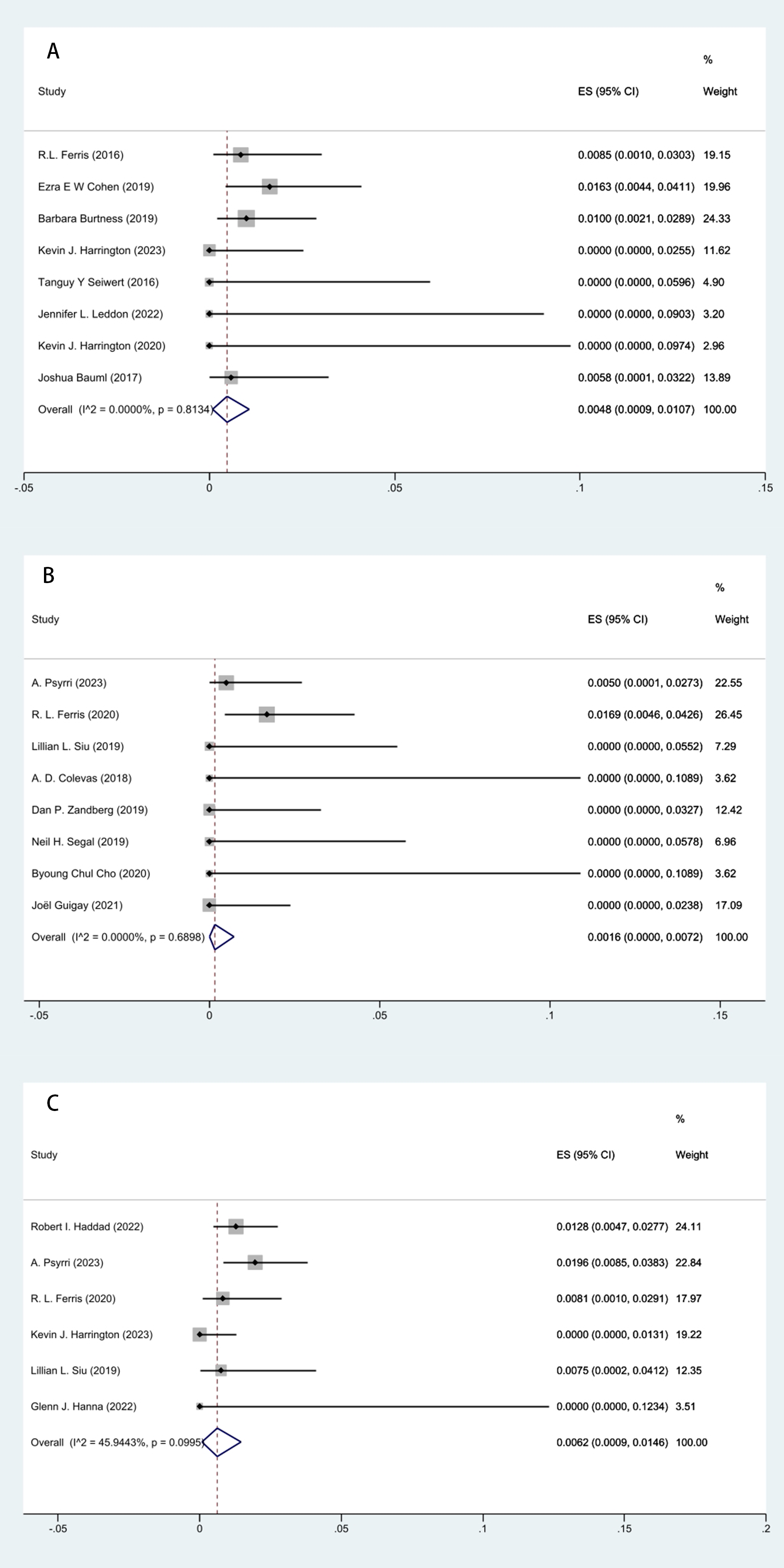


Figure S4. Pooled incidences of treatment-related deaths: patients treated with PD-1 (A), patients treated with PD-L1 (B), patients treated with combination therapy (C).


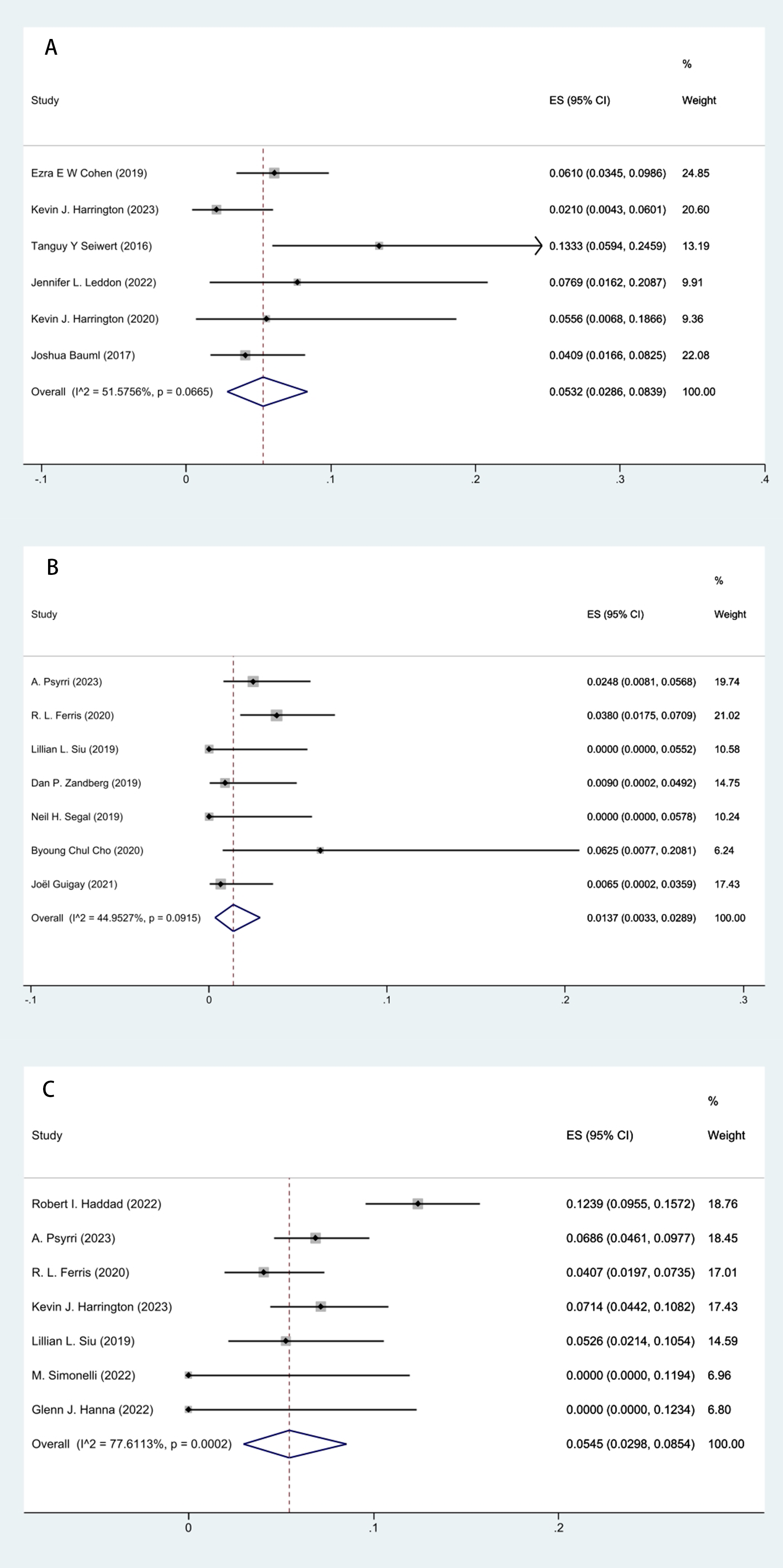


Figure S5. Pooled incidences of trAEs leading to discontinuation of treatment: patients treated with PD-1 (A), patients treated with PD-L1 (B), patients treated with combination therapy (C).
