# Supplementary material for: Toxicity profiles of immune checkpoint inhibitors for recurrent or metastatic head and neck squamous cell carcinoma: A systematic review and meta‐analysis
Source: Cancer Med. 2024 Mar 30;13(7):e7119. doi: 10.1002/cam4.7119 (PMC10980932; doi:10.1002/cam4.7119)
Supplement: Supplementary file 2 — Data S2. [file CAM4-13-e7119-s002.docx]

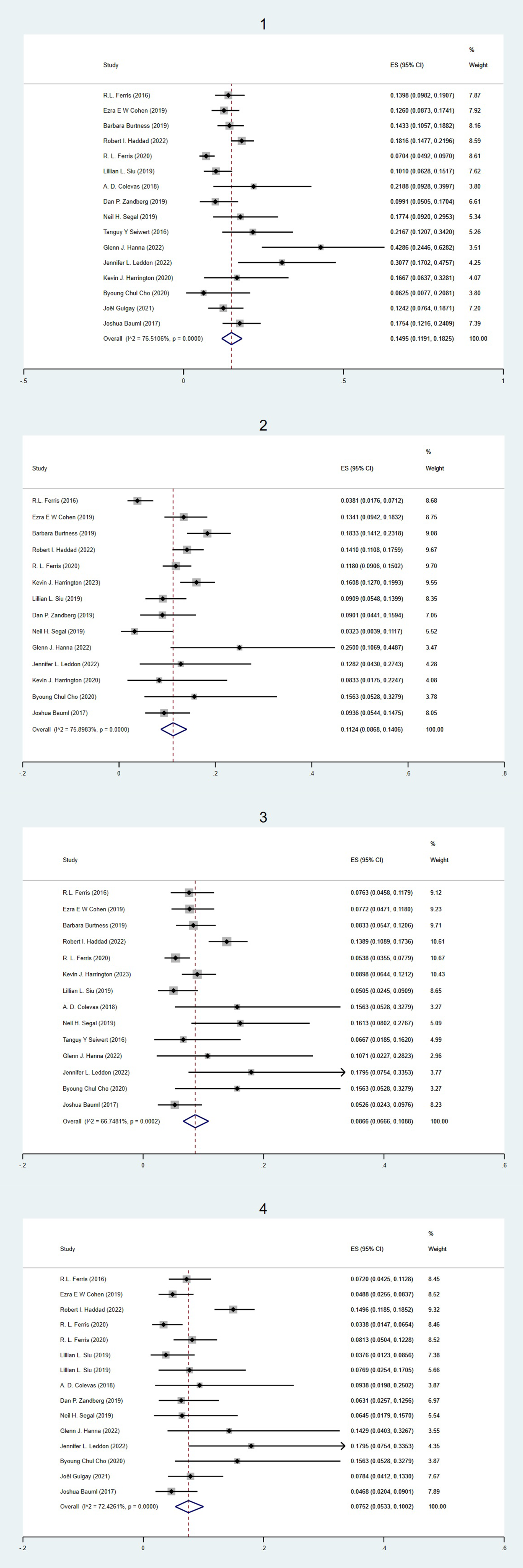


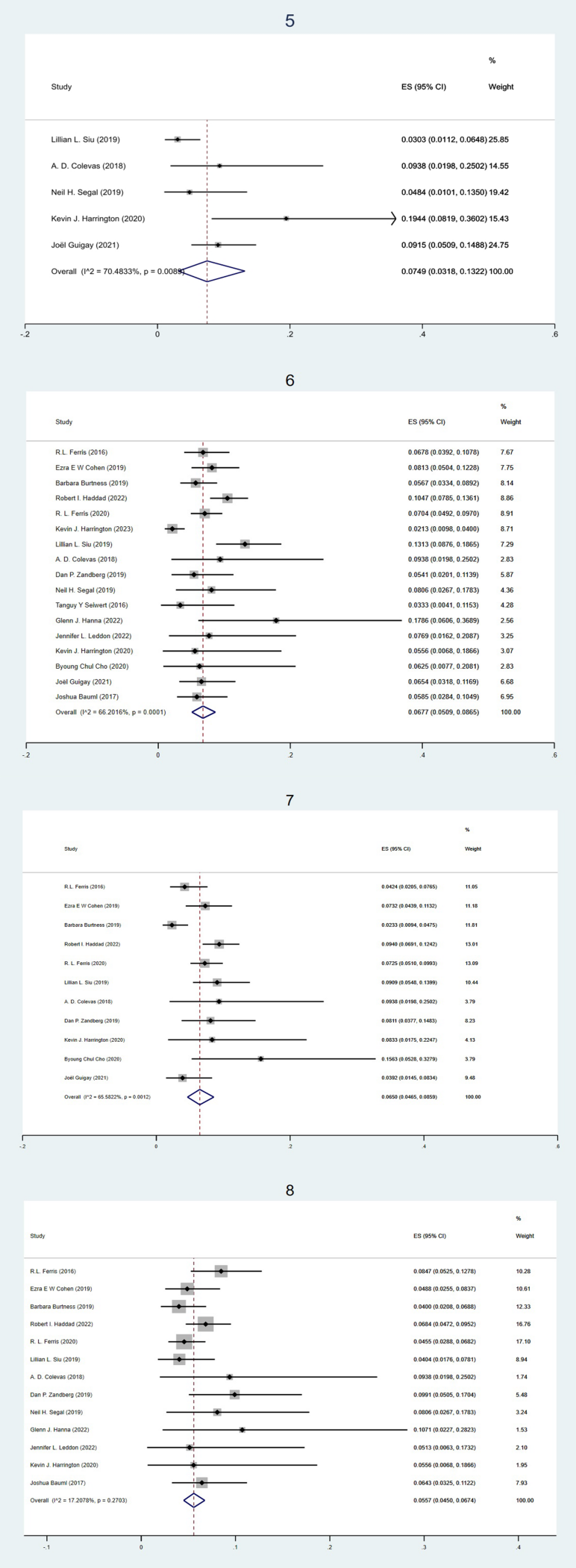


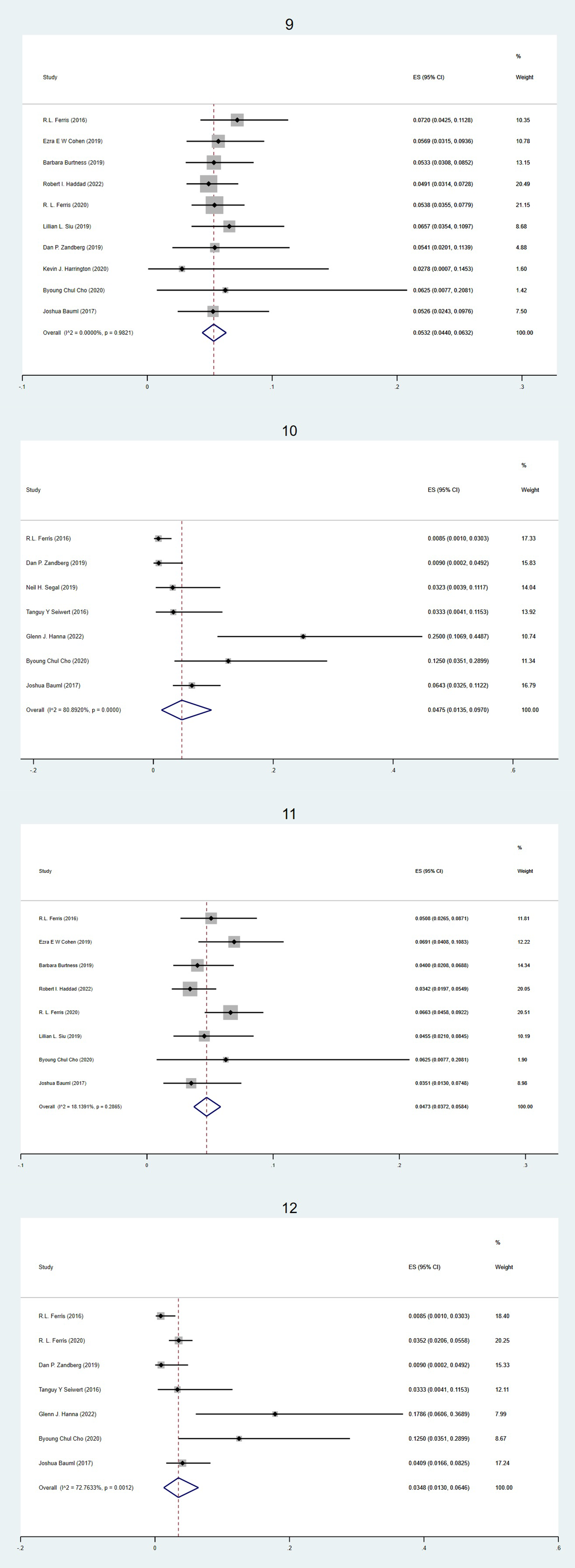


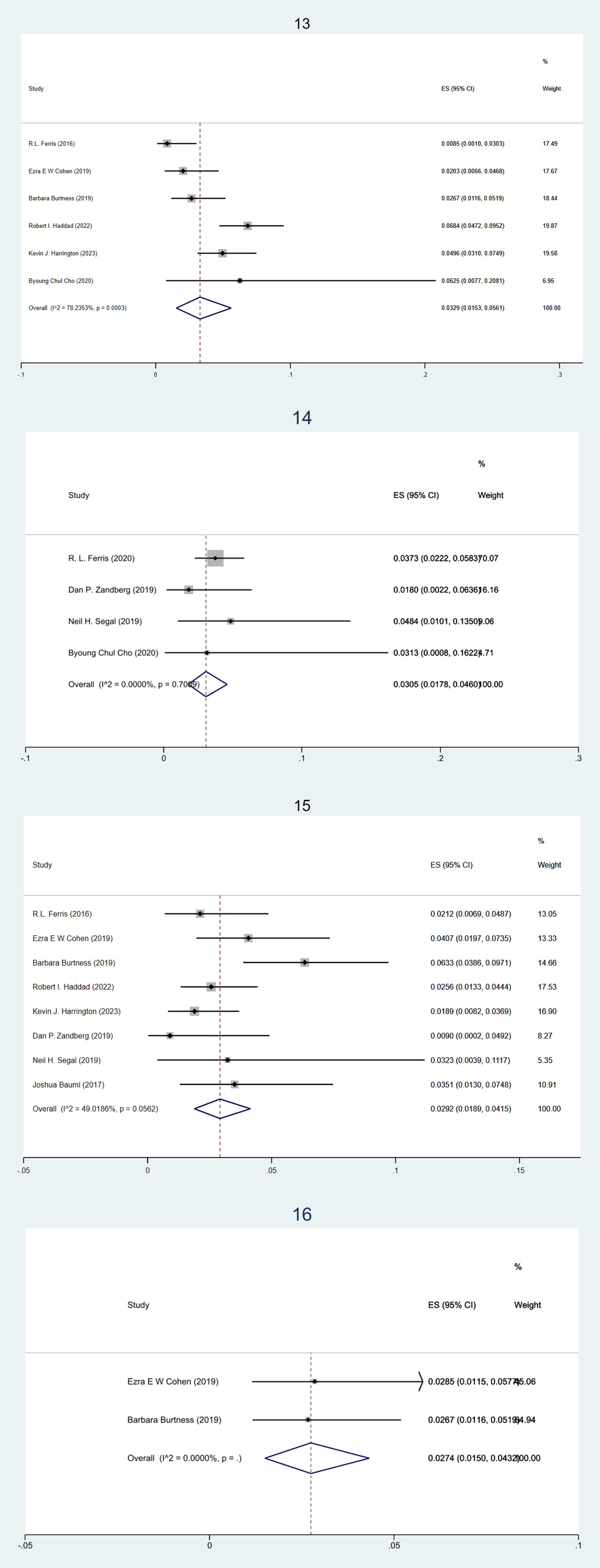


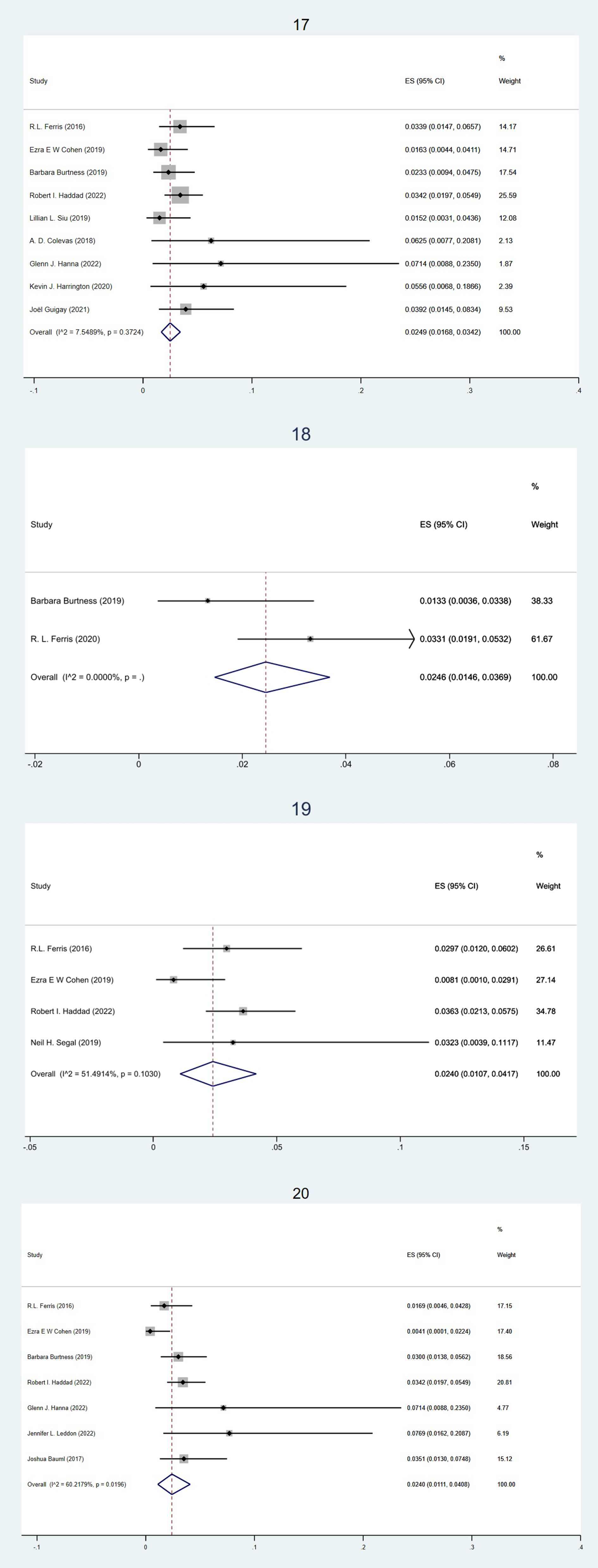


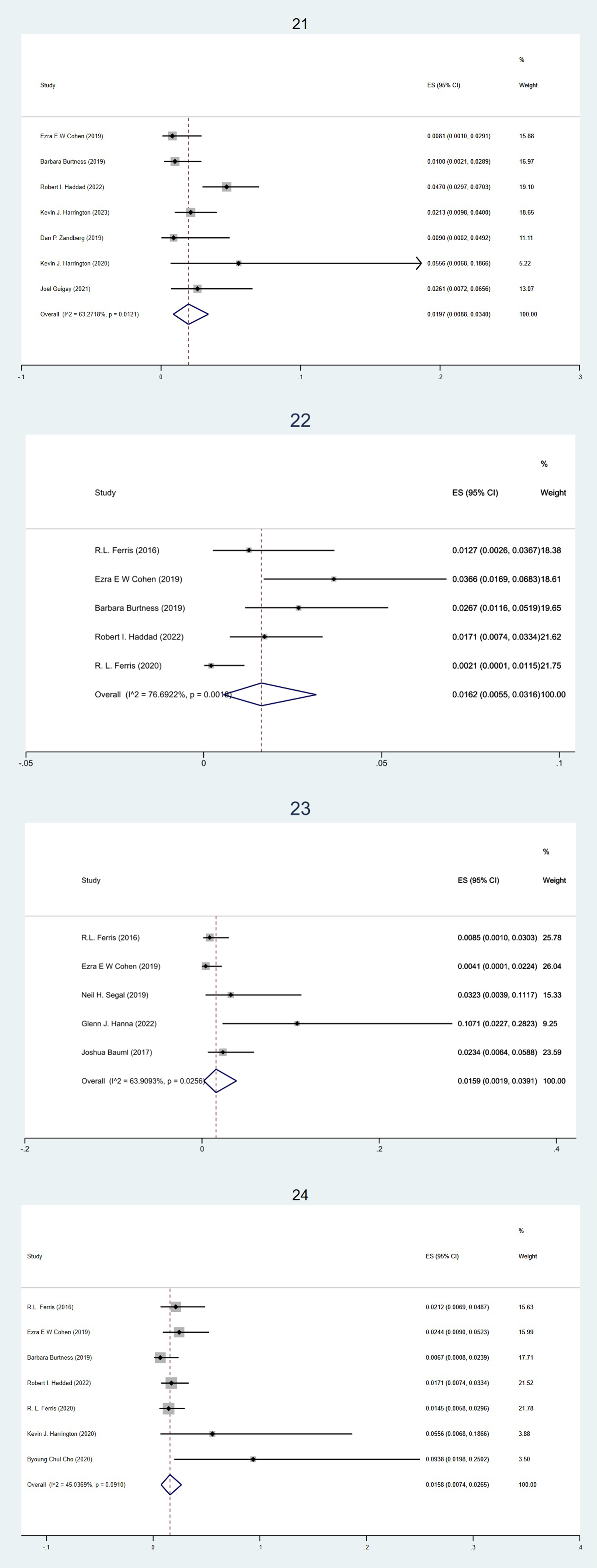


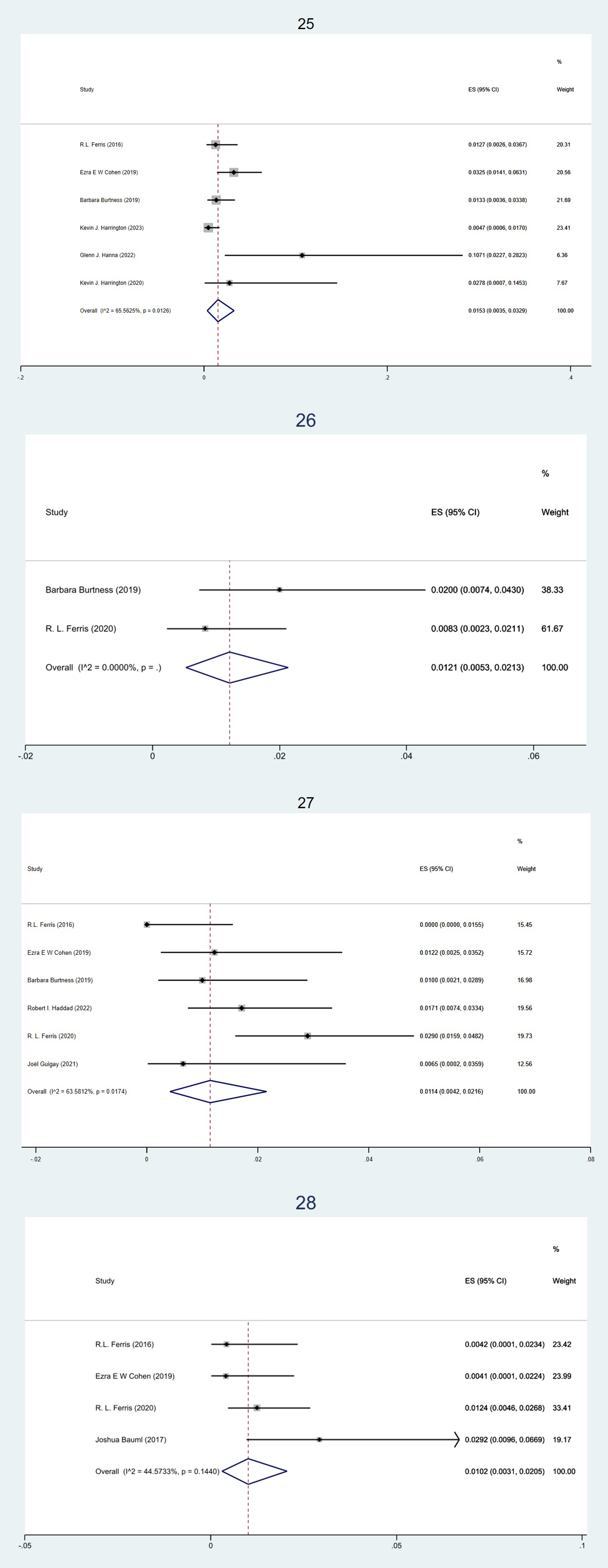


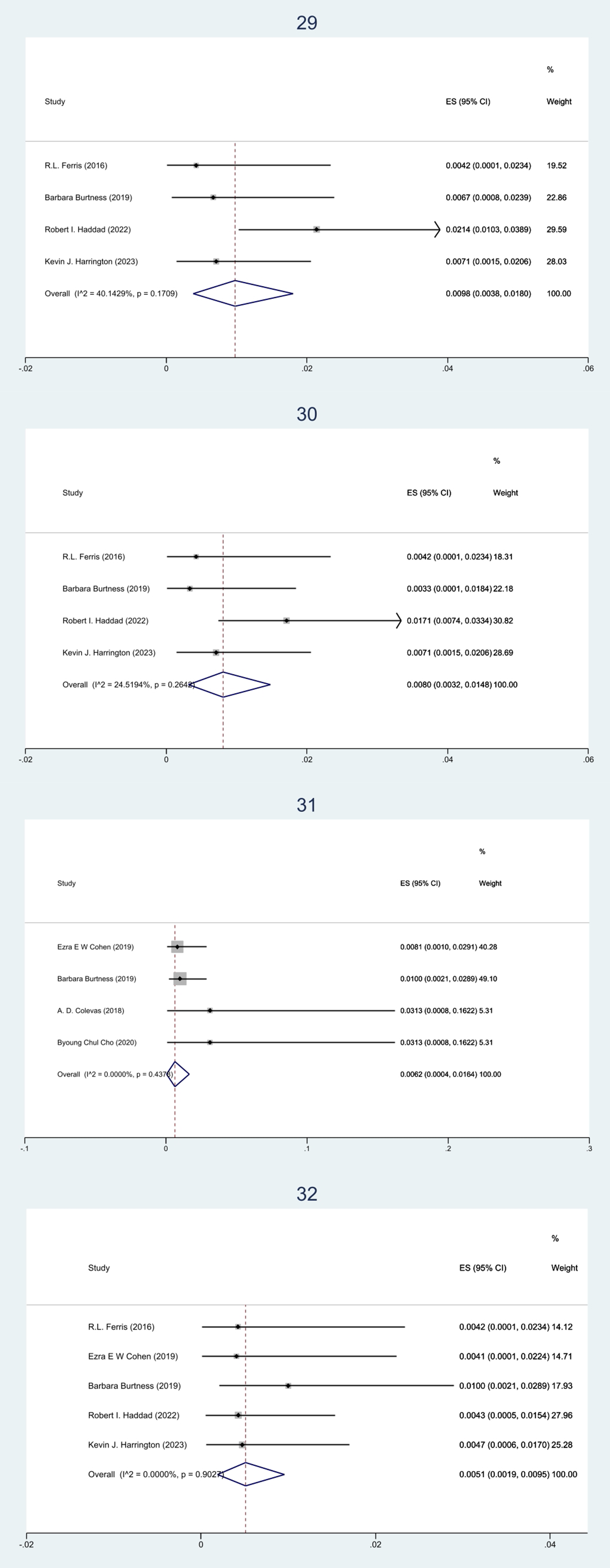


Figure S6. Pooled incidences of any grade specific trAEs: fatigue (1), hypothyroidism (2), rash (3), pruritus (4), pyrexia (5), diarrhea (6), asthenia (7), nausea (8), decreased appetite (9), AST increased (10), anemia (11), ALT increased (12), hyperthyroidism (13), elevated GGT (14), pneumonitis (15), severe skin reaction (16), vomiting (17), thrombocytopenia (18), dry skin (19), weight loss (20), hepatitis (21), mucosal inflammation (22), blood alkaline phosphatase increased (23), stomatitis (24), infusion-related reaction (25), dermatitis acneiform (26), neutrophil count decreased (27), peripheral neuropathy (28), adrenocortical insufficiency (29), hypophysitis (30), colitis (31), acute kidney injury/nephritis (32).
