# Supplementary material for: Toxicity profiles of immune checkpoint inhibitors for recurrent or metastatic head and neck squamous cell carcinoma: A systematic review and meta‐analysis
Source: Cancer Med. 2024 Mar 30;13(7):e7119. doi: 10.1002/cam4.7119 (PMC10980932; doi:10.1002/cam4.7119)
Supplement: Supplementary file 3 — Data S3. [file CAM4-13-e7119-s001.docx]

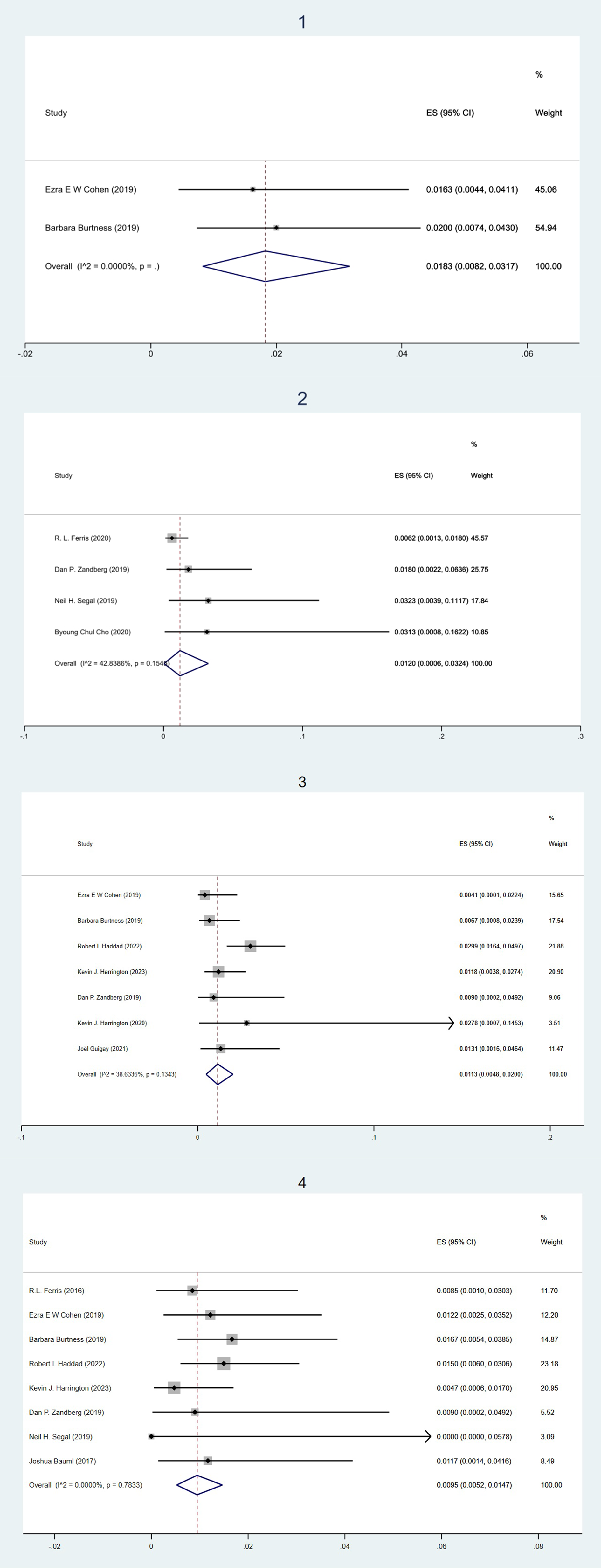


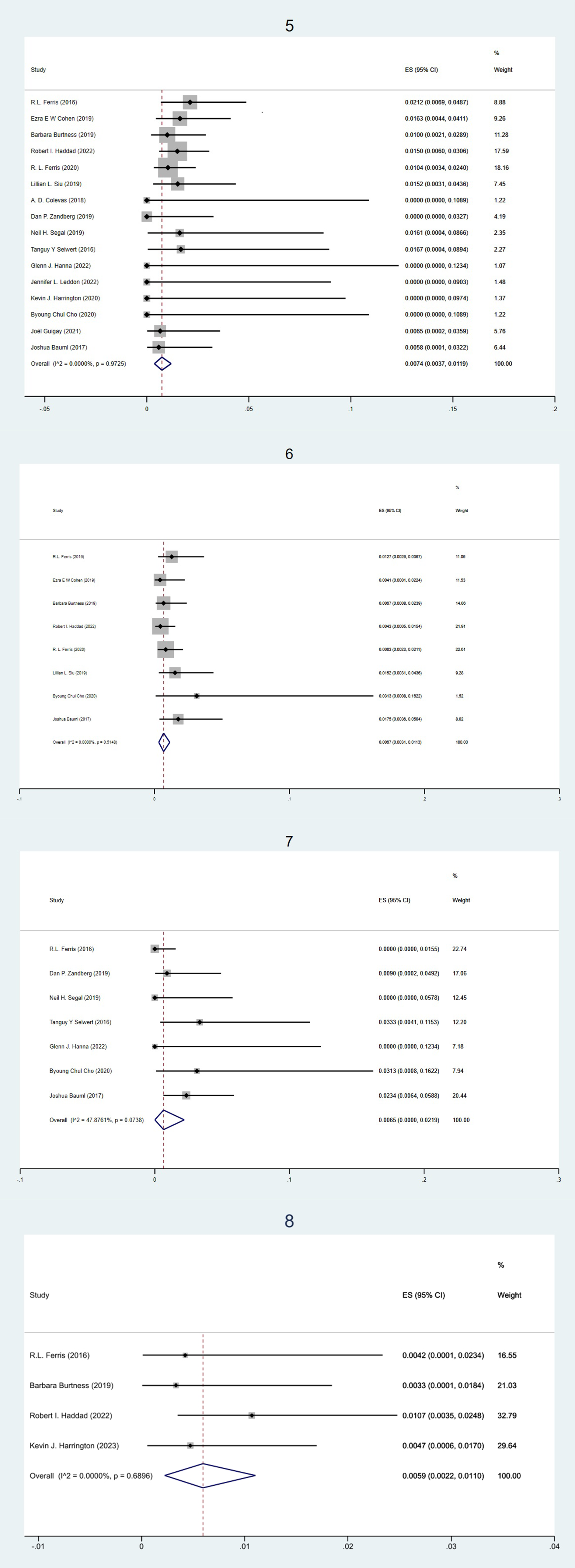


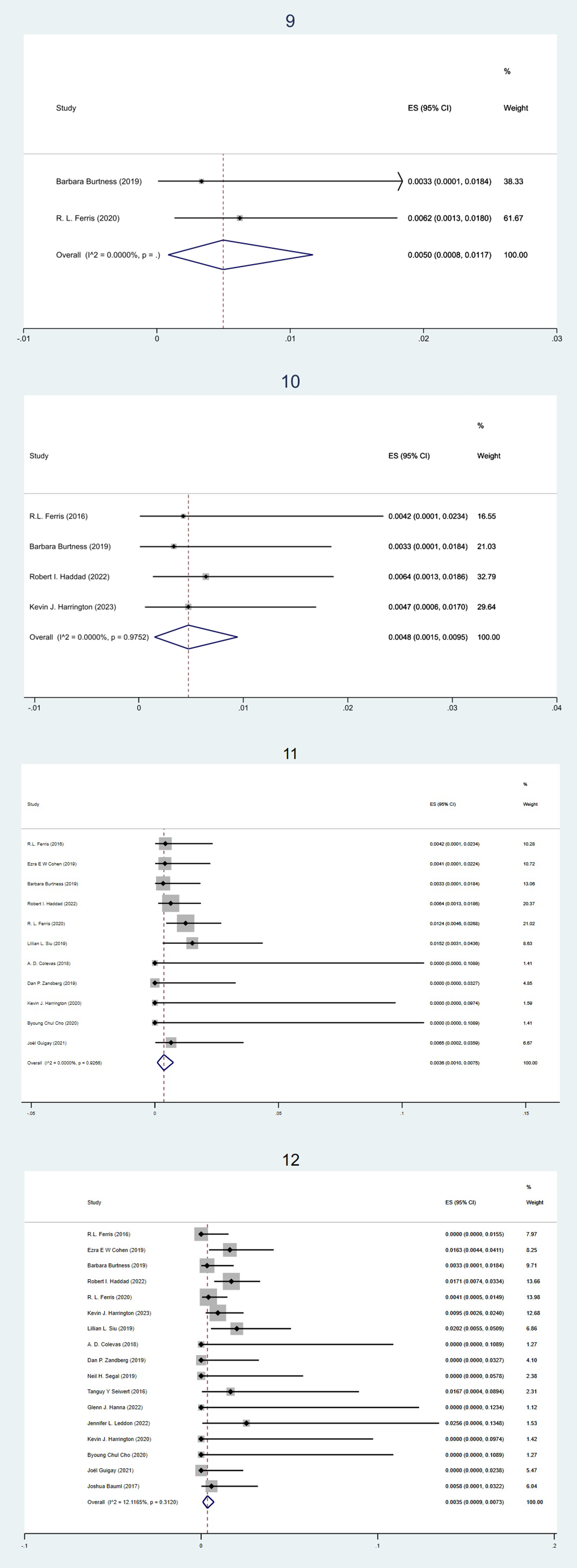


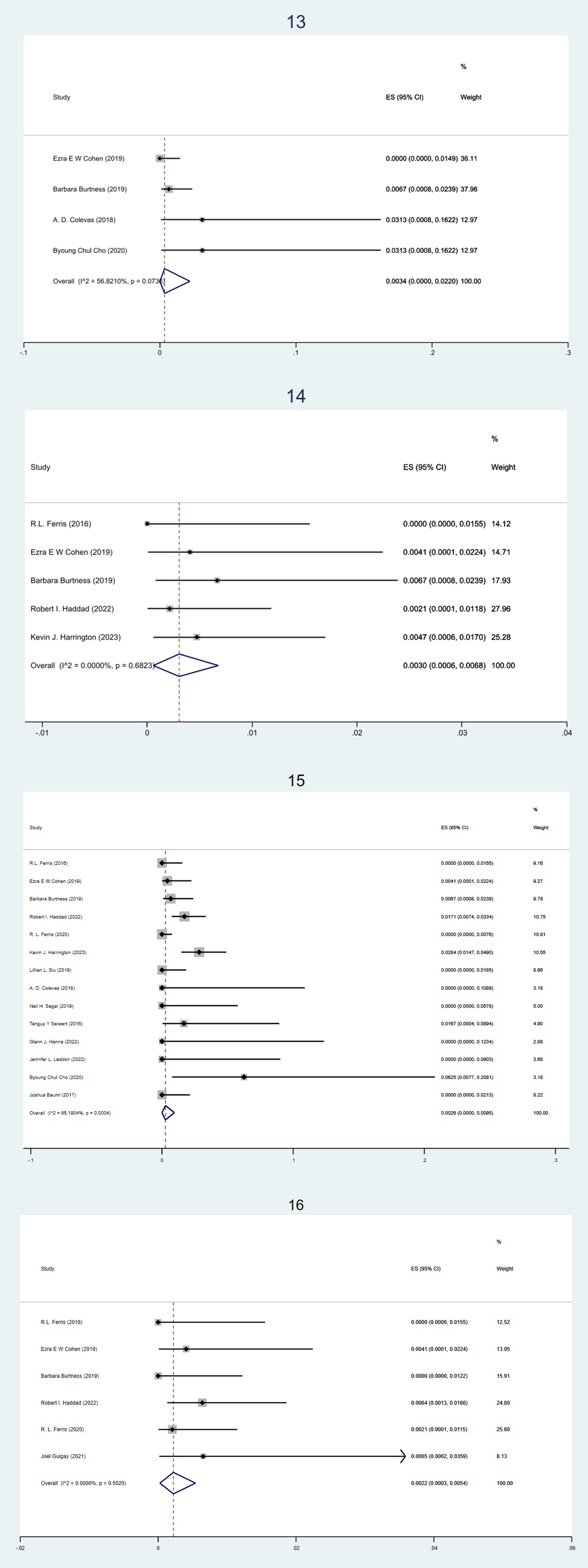


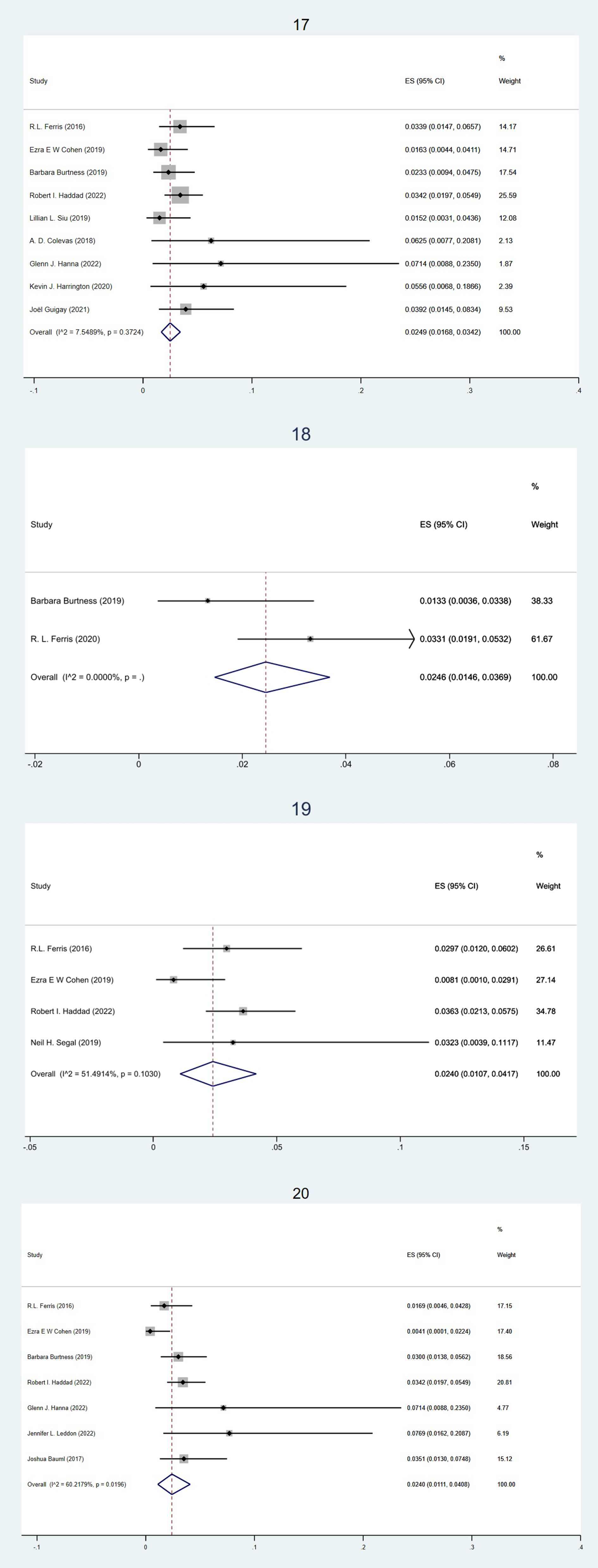


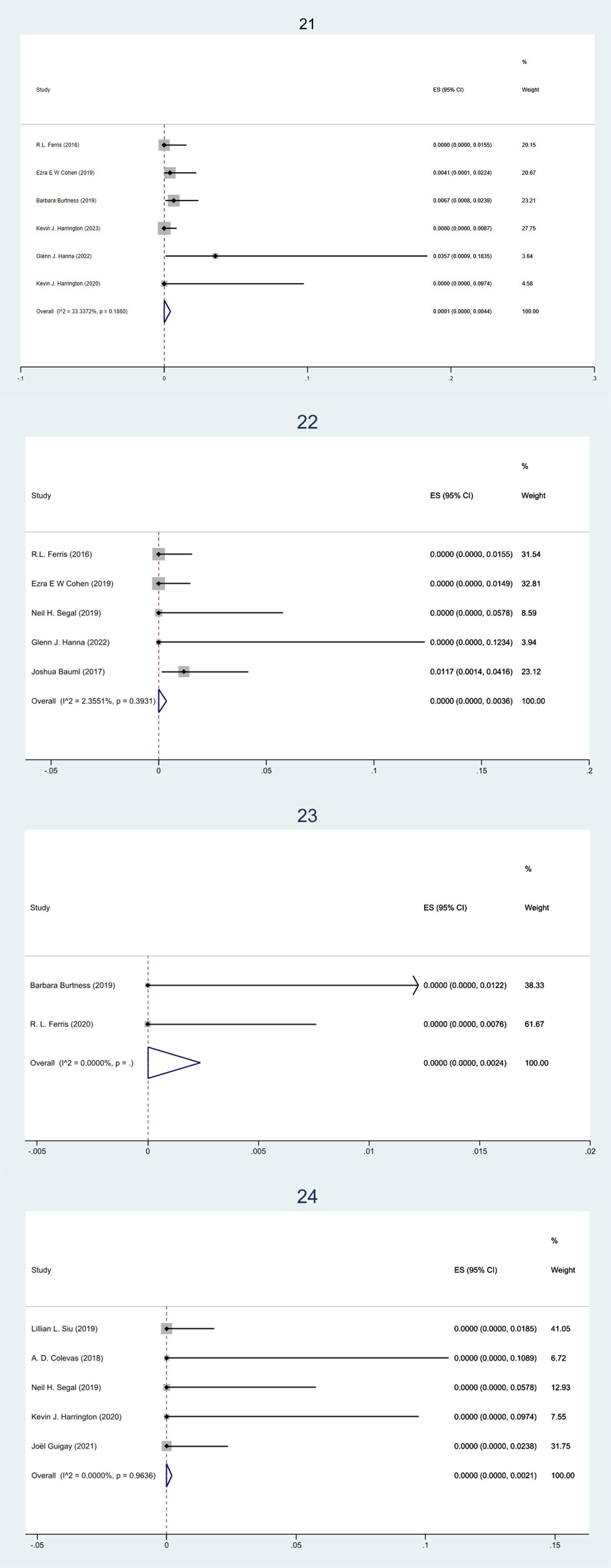


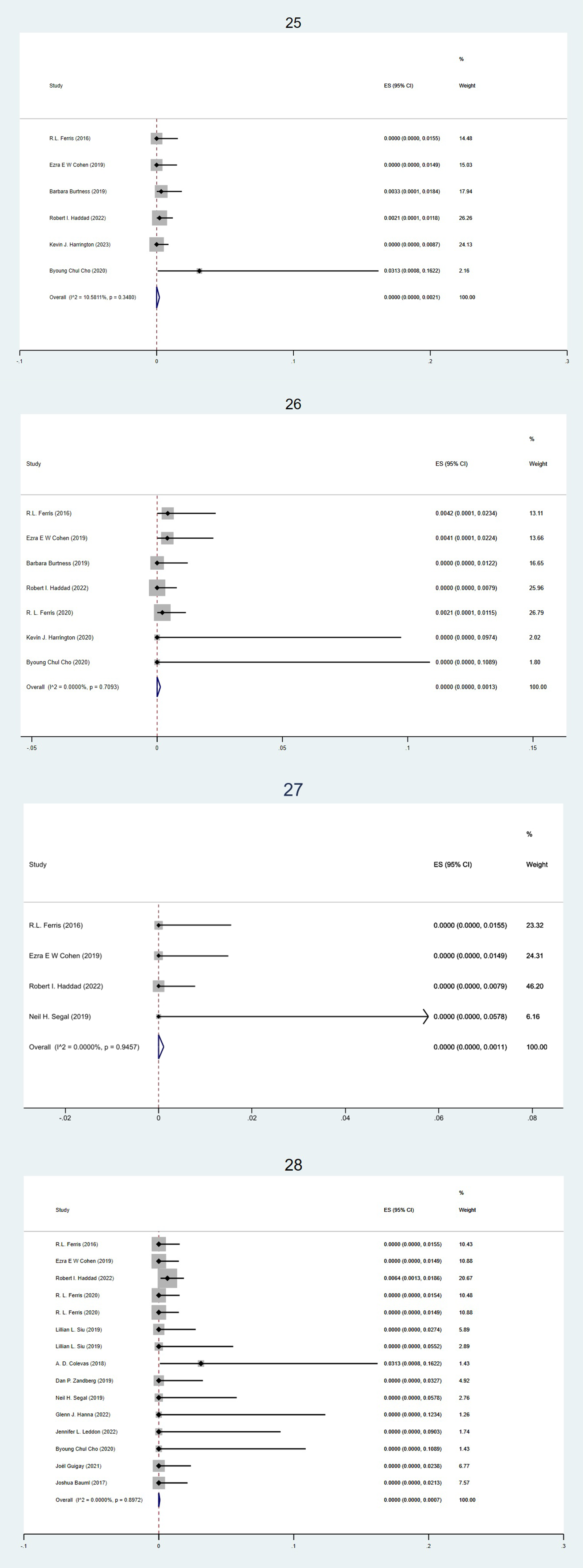


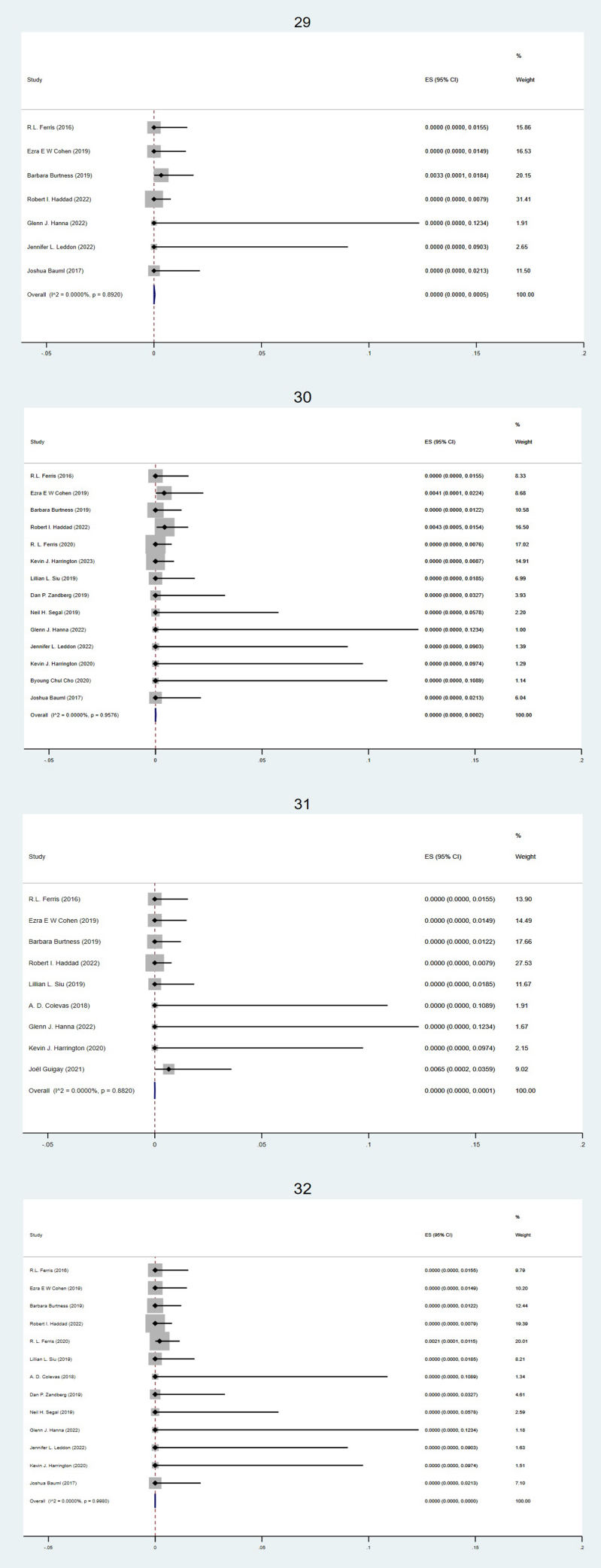


Figure S7. Pooled incidences of any grade 3 or more specific trAEs: severe skin reaction (1), elevated GGT (2), hepatitis (3), pneumonitis (4), fatigue (5), anemia (6), AST increased (7), hypophysitis (8), thrombocytopenia (9), adrenocortical insufficiency (10), asthenia (11), diarrhea (12), colitis (13), acute kidney injury/nephritis (14), rash (15), neutrophil count decreased (16), ALT increased (17), mucosal inflammation (18), peripheral neuropathy (19), decreased appetite (20), infusion-related reaction (21), blood alkaline phosphatase increased (22), dermatitis acneiform (23), pyrexia (24), hyperthyroidism (25), stomatitis (26), dry skin (27), pruritus (28), weight loss (29), hypothyroidism (30), vomiting (31), nausea (32).
